# Supplementary figures and images for: Protease-mediated PRC1 dissociation promotes H2AK119ub remodeling during stress responses
Source: EMBO J. 2026 Mar 11;45(8):2561–86. doi: 10.1038/s44318-026-00729-9 (PMC13083880; doi:10.1038/s44318-026-00729-9)

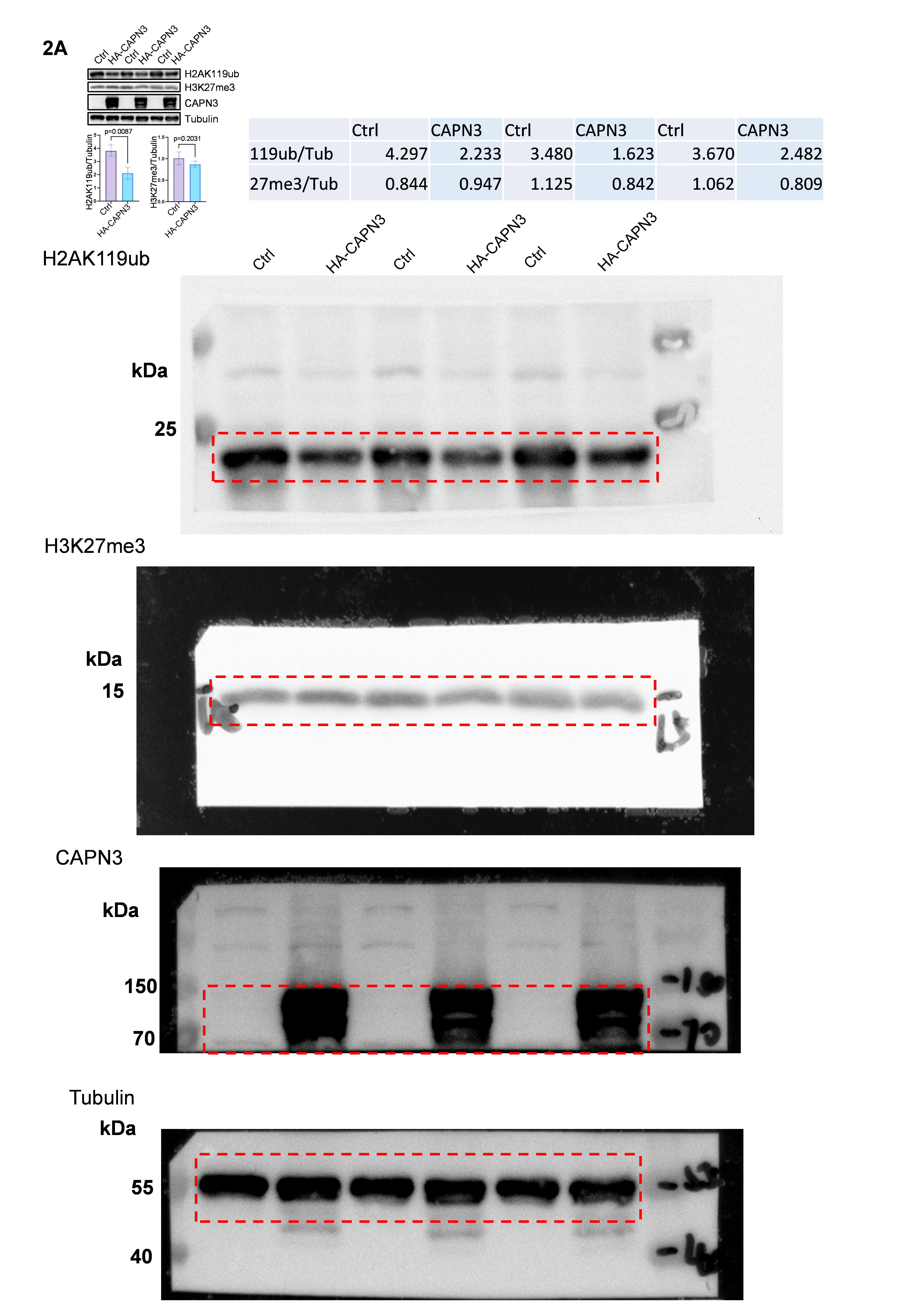

Supplement: Supplementary file 4 — Source data Fig. 2 [file 44318_2026_729_MOESM4_ESM.zip › fig2/2A.tif]

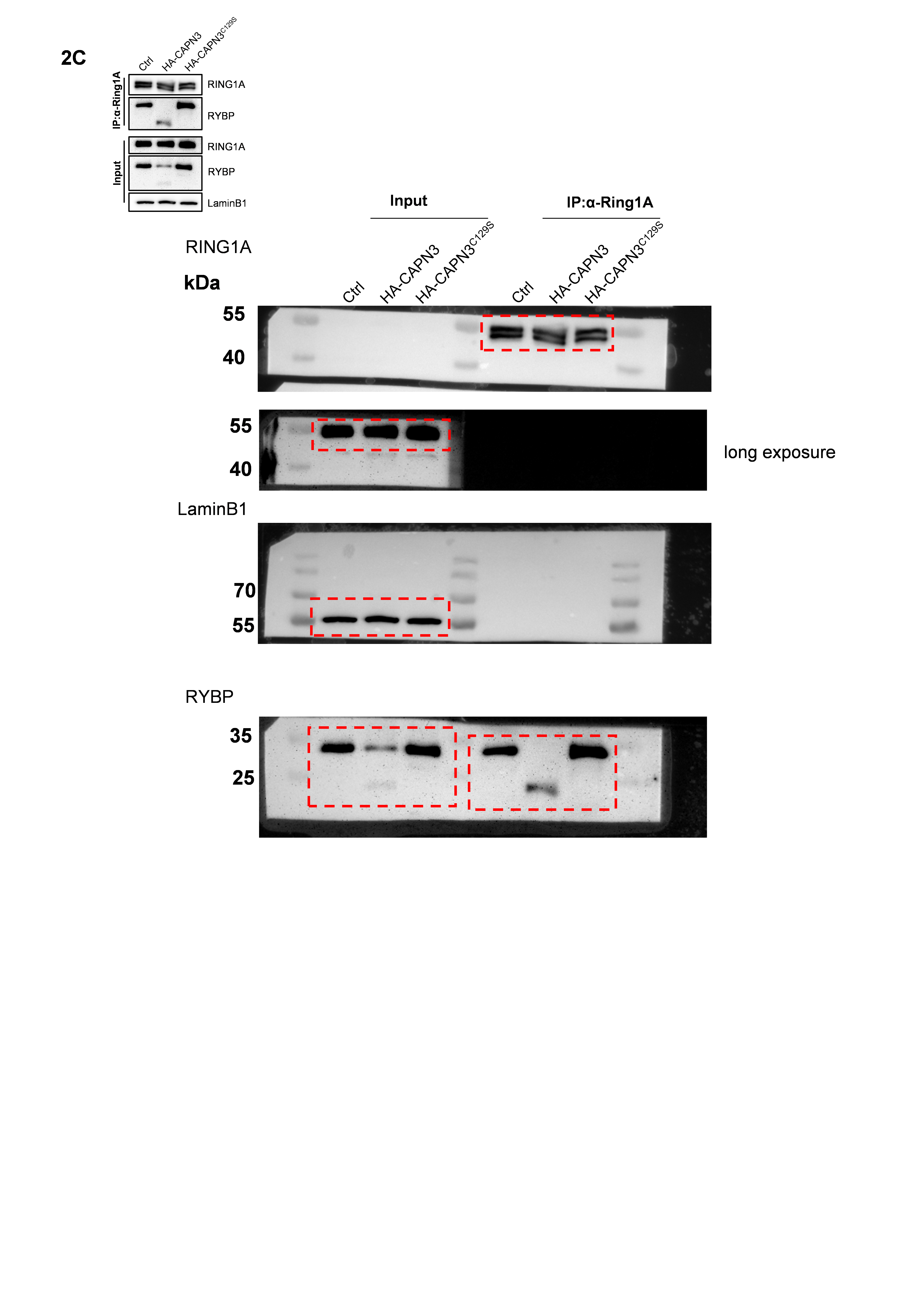

Supplement: Supplementary file 4 — Source data Fig. 2 [file 44318_2026_729_MOESM4_ESM.zip › fig2/2C.tif]

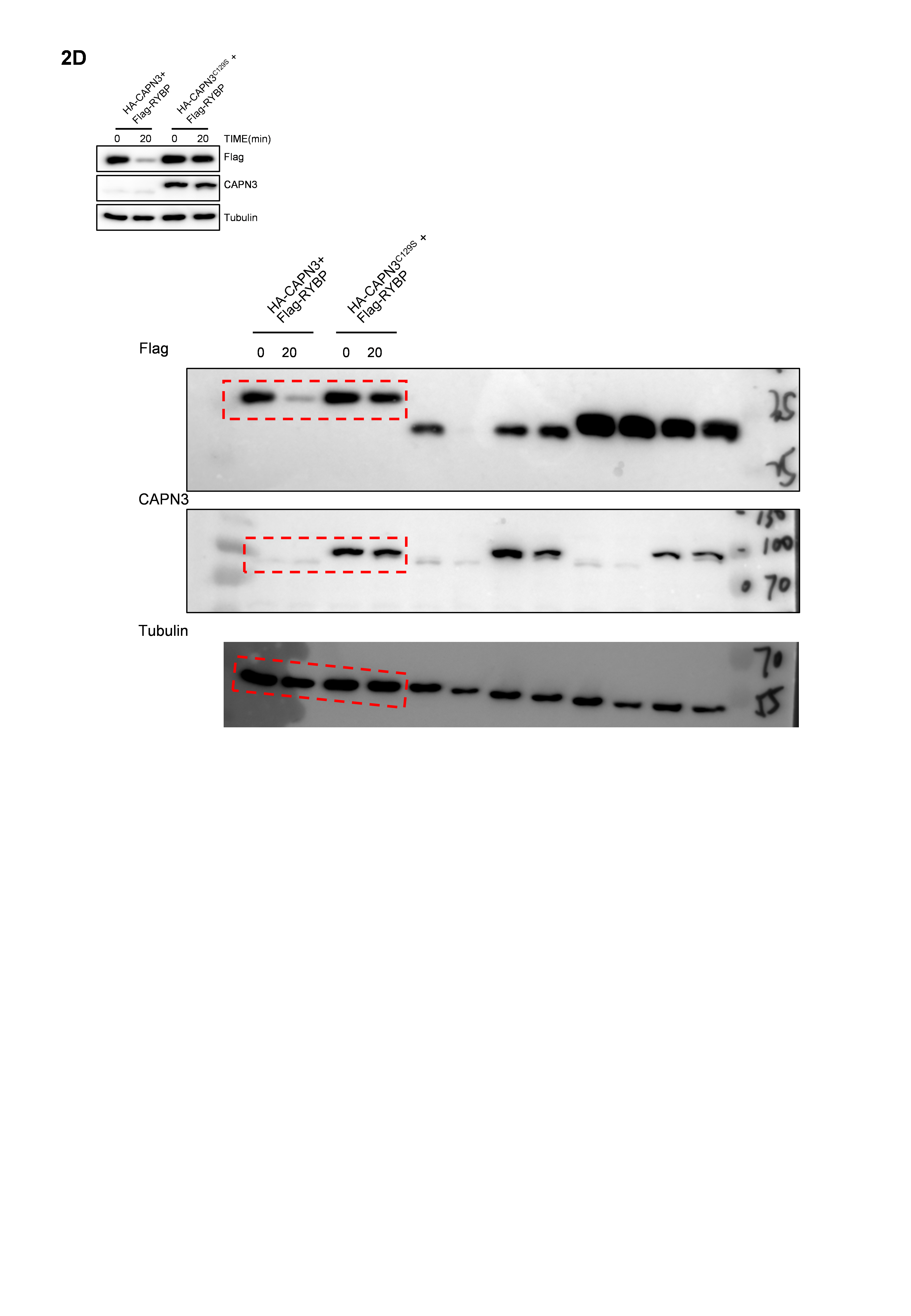

Supplement: Supplementary file 4 — Source data Fig. 2 [file 44318_2026_729_MOESM4_ESM.zip › fig2/2D.tif]

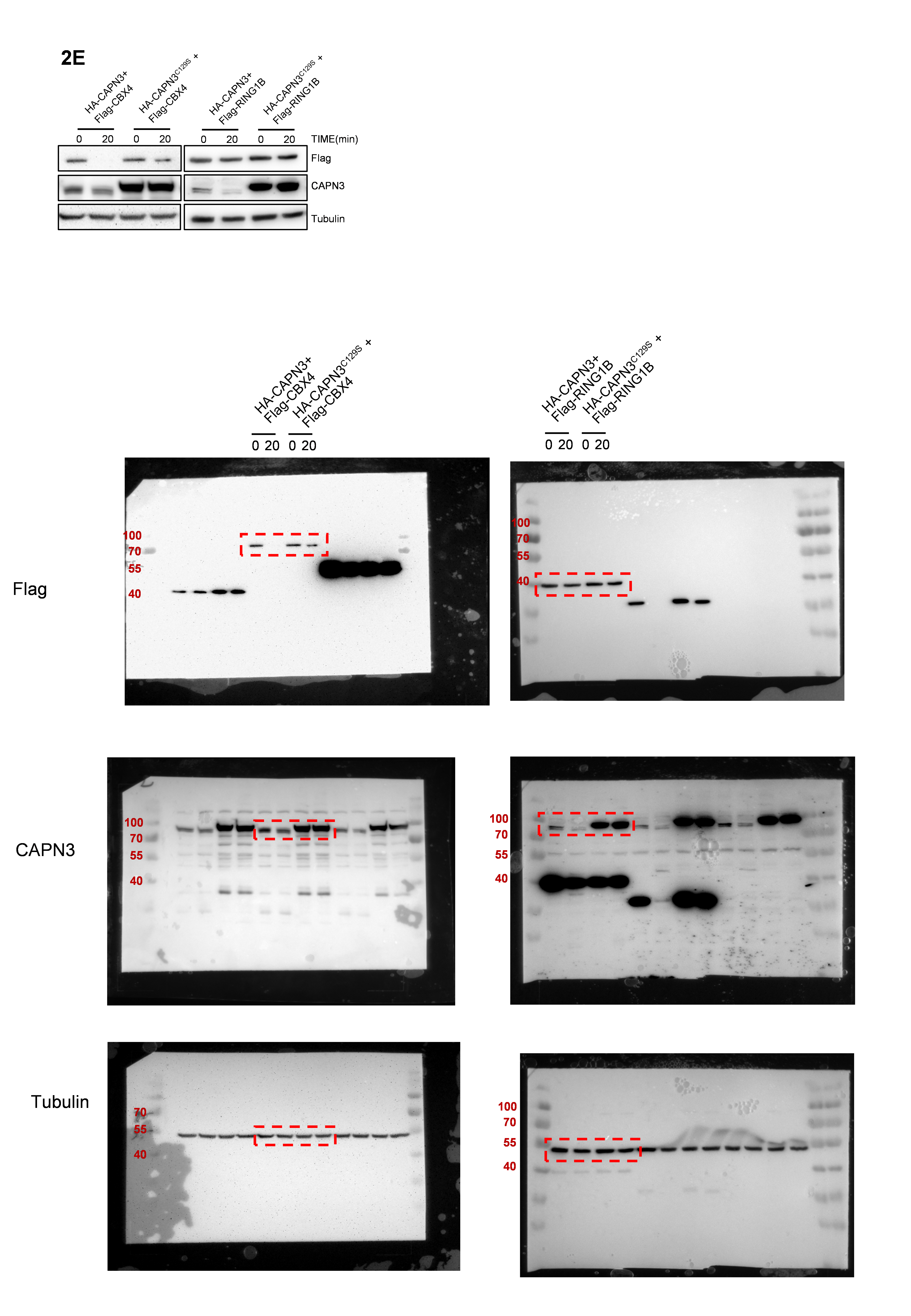

Supplement: Supplementary file 4 — Source data Fig. 2 [file 44318_2026_729_MOESM4_ESM.zip › fig2/2E.tif]

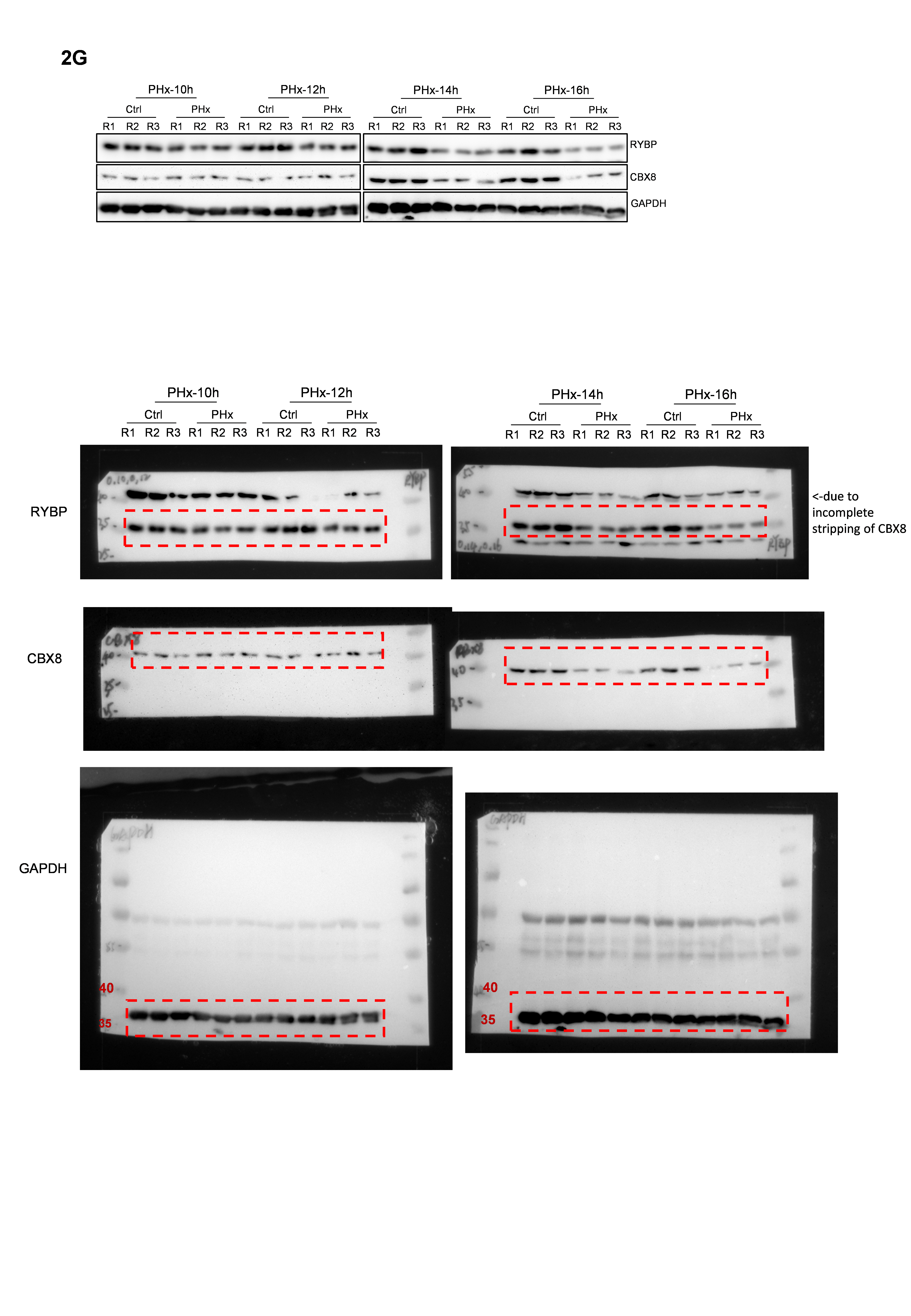

Supplement: Supplementary file 4 — Source data Fig. 2 [file 44318_2026_729_MOESM4_ESM.zip › fig2/2G.tif]

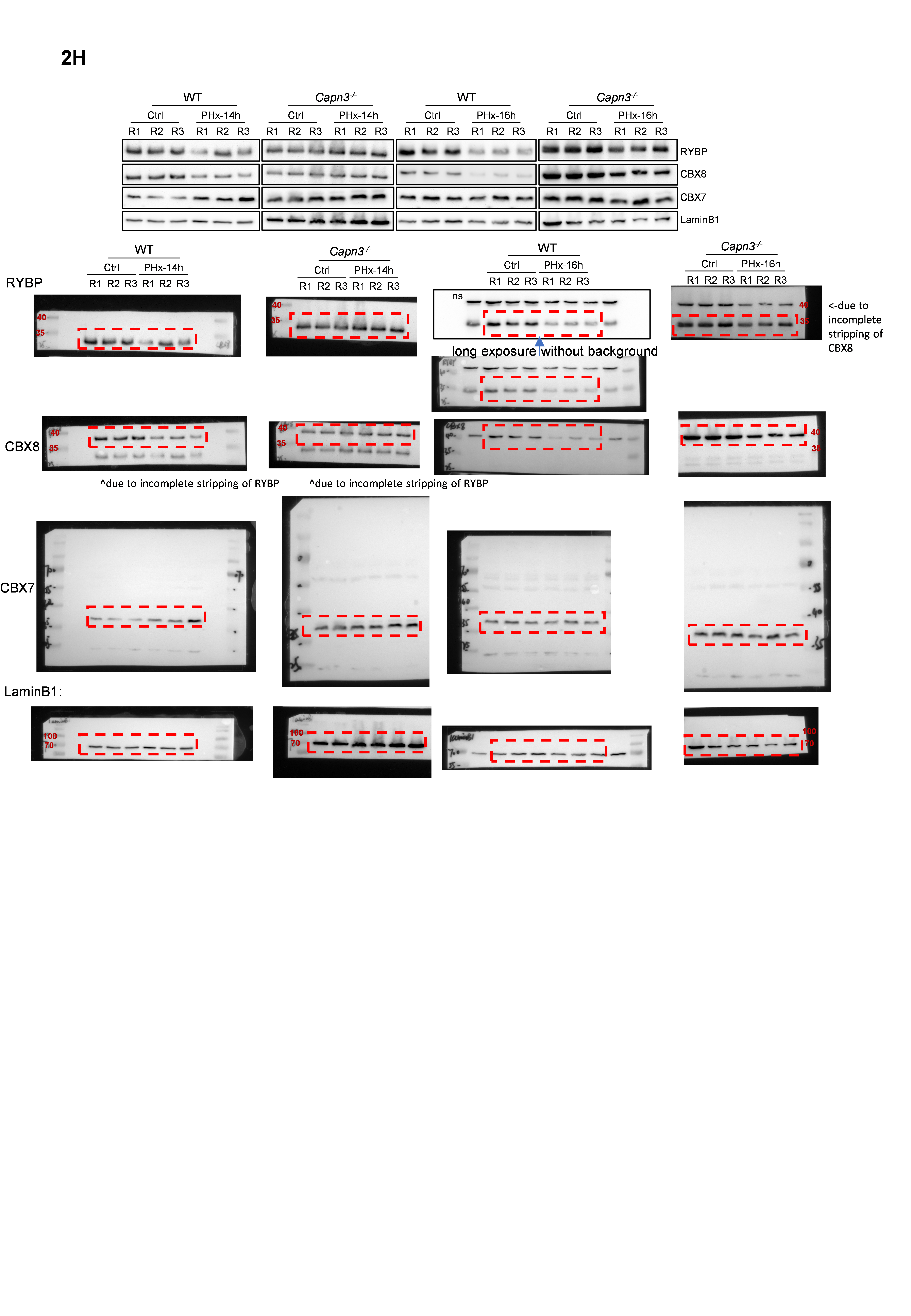

Supplement: Supplementary file 4 — Source data Fig. 2 [file 44318_2026_729_MOESM4_ESM.zip › fig2/2H.tif]

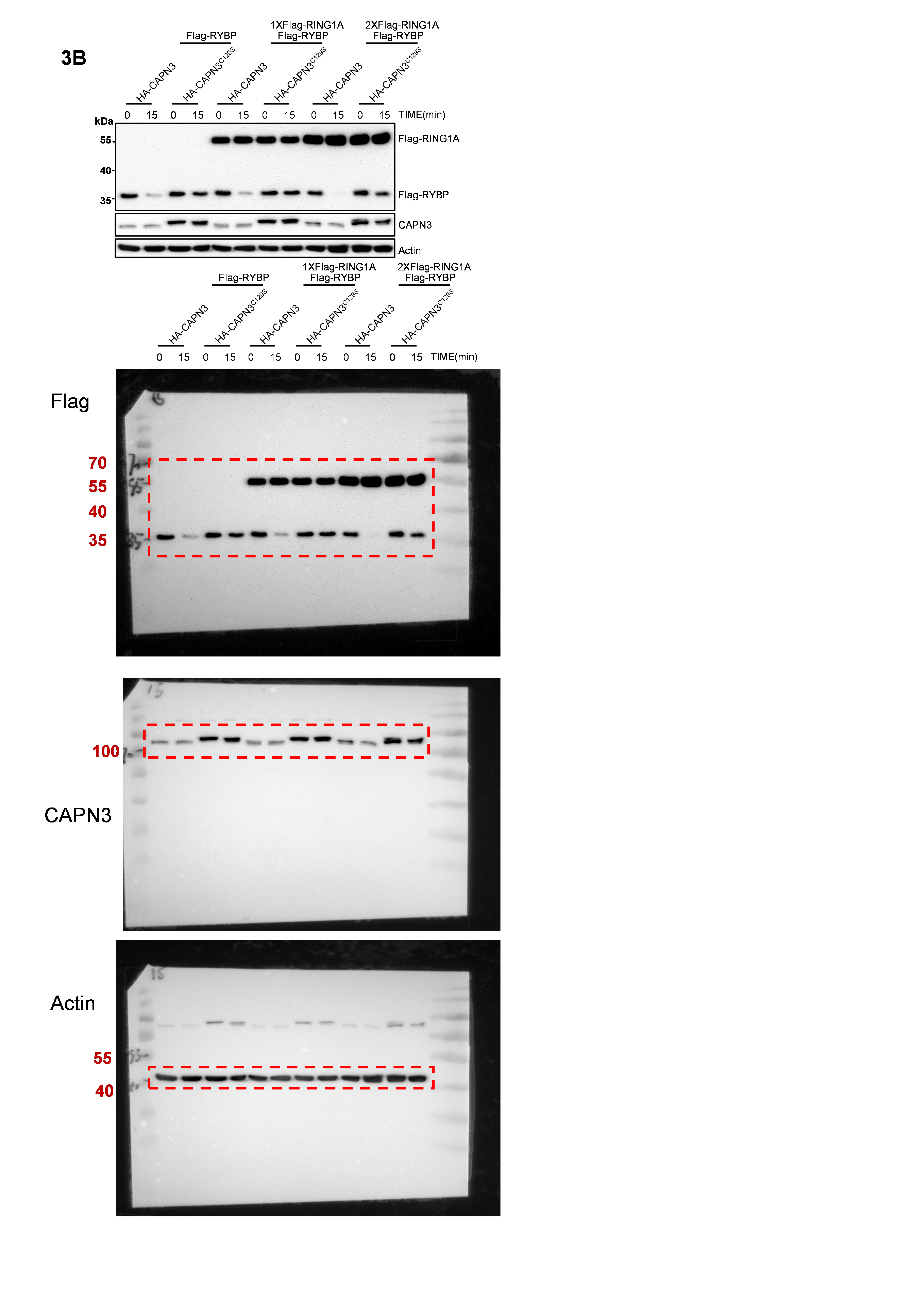

Supplement: Supplementary file 5 — Source data Fig. 3 [file 44318_2026_729_MOESM5_ESM.zip › fig3/3B.tif]

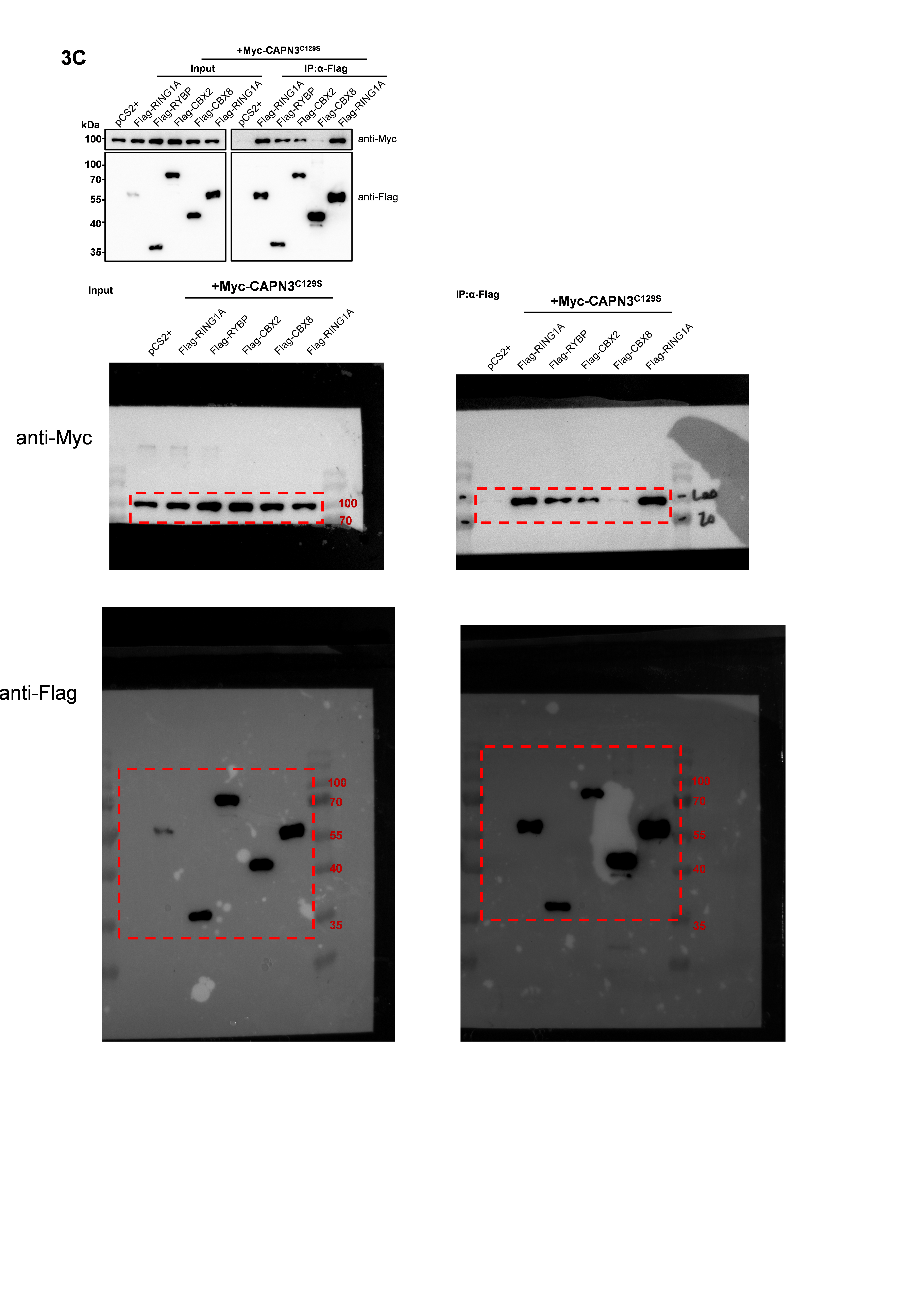

Supplement: Supplementary file 5 — Source data Fig. 3 [file 44318_2026_729_MOESM5_ESM.zip › fig3/3C.tif]

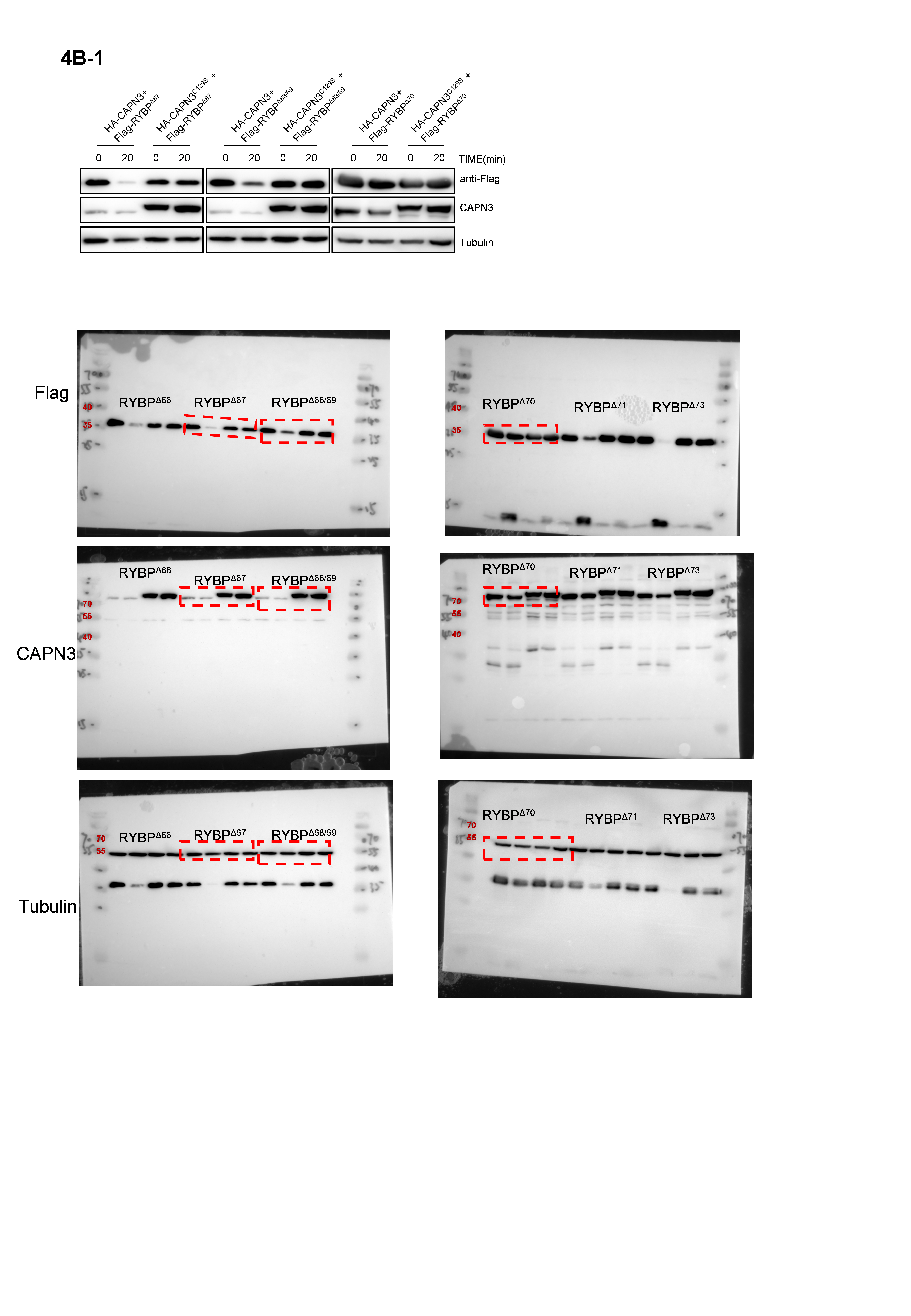

Supplement: Supplementary file 6 — Source data Fig. 4 [file 44318_2026_729_MOESM6_ESM.zip › fig4/4B-1.tif]

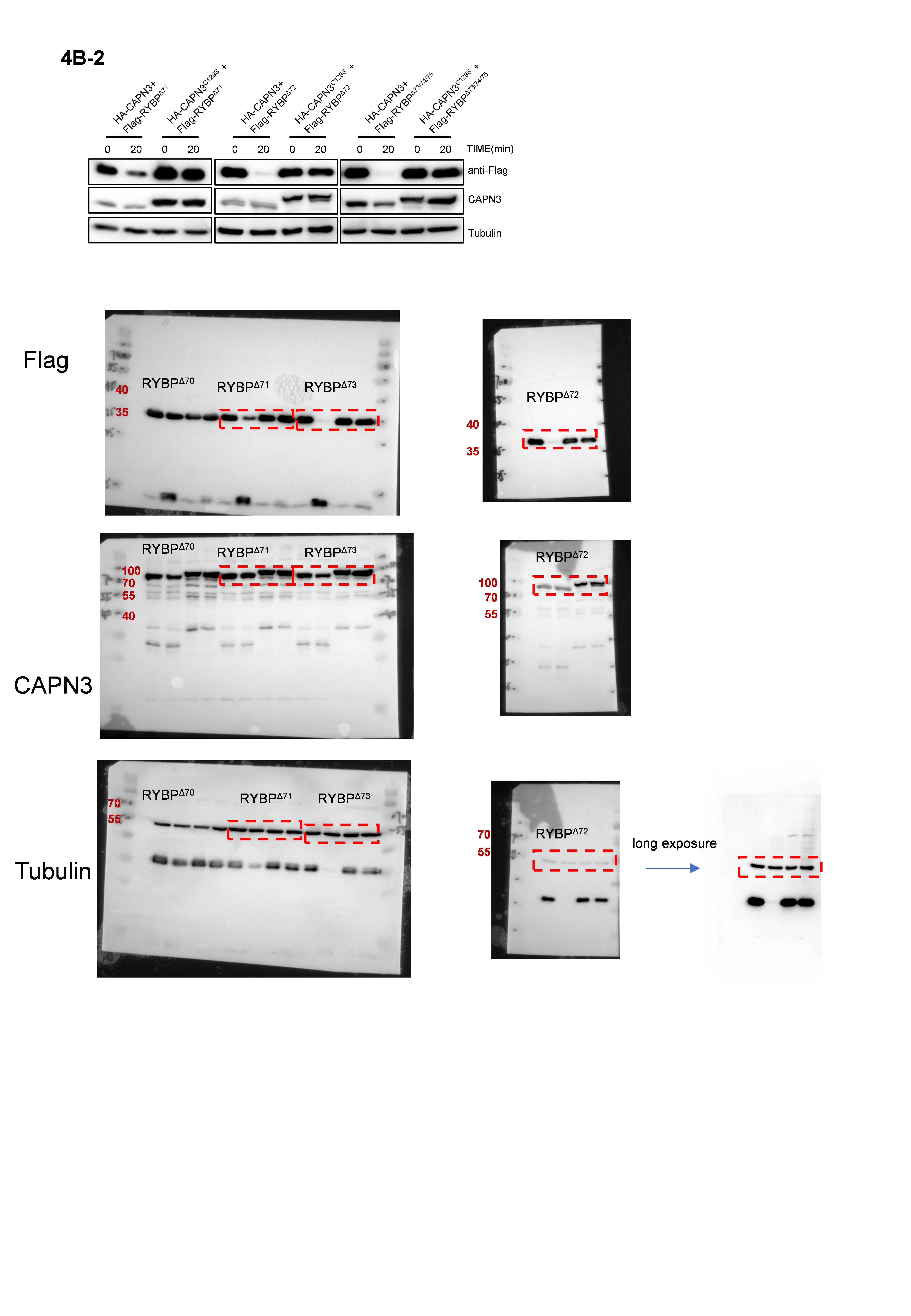

Supplement: Supplementary file 6 — Source data Fig. 4 [file 44318_2026_729_MOESM6_ESM.zip › fig4/4B-2.tif]

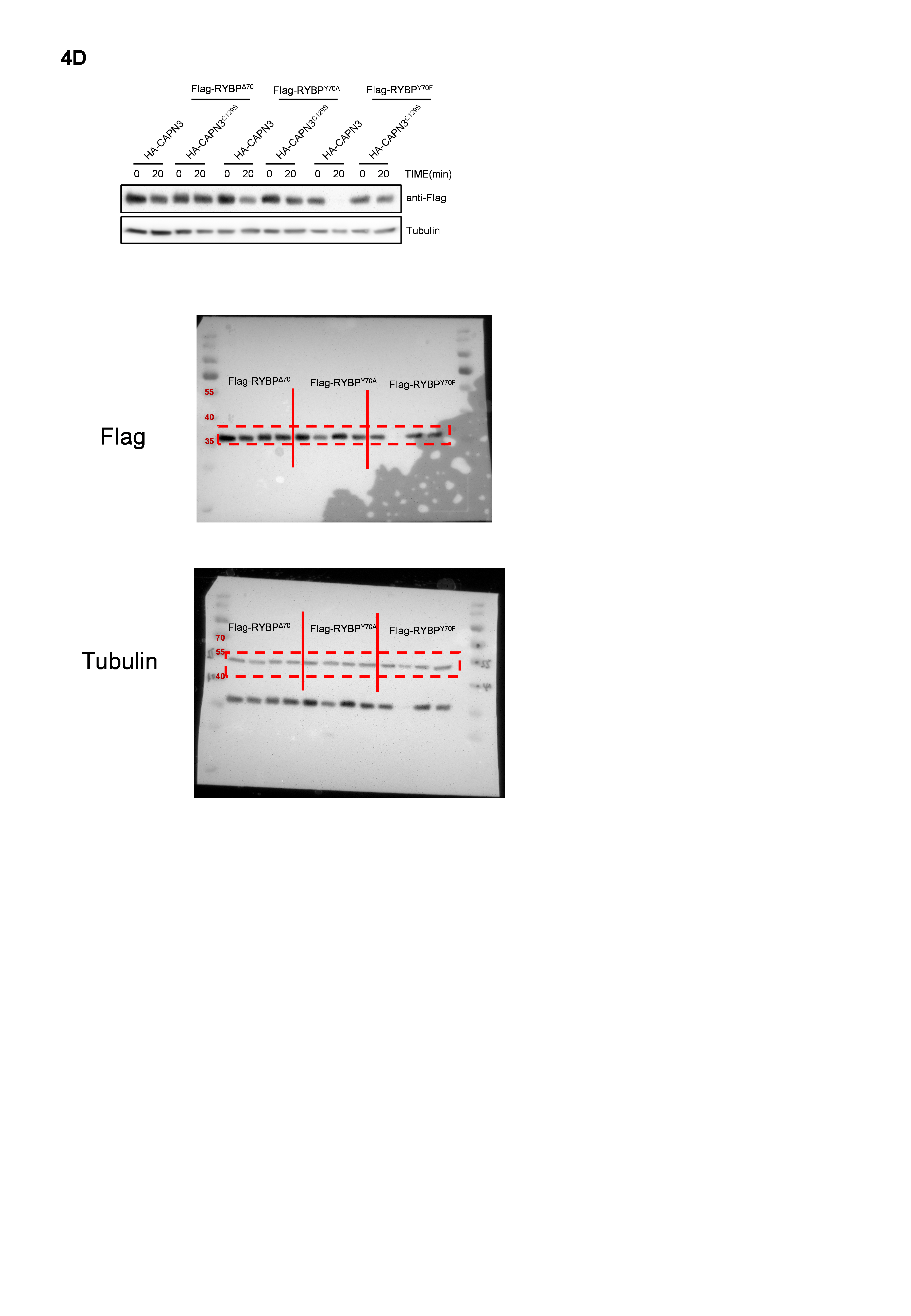

Supplement: Supplementary file 6 — Source data Fig. 4 [file 44318_2026_729_MOESM6_ESM.zip › fig4/4D.tif]

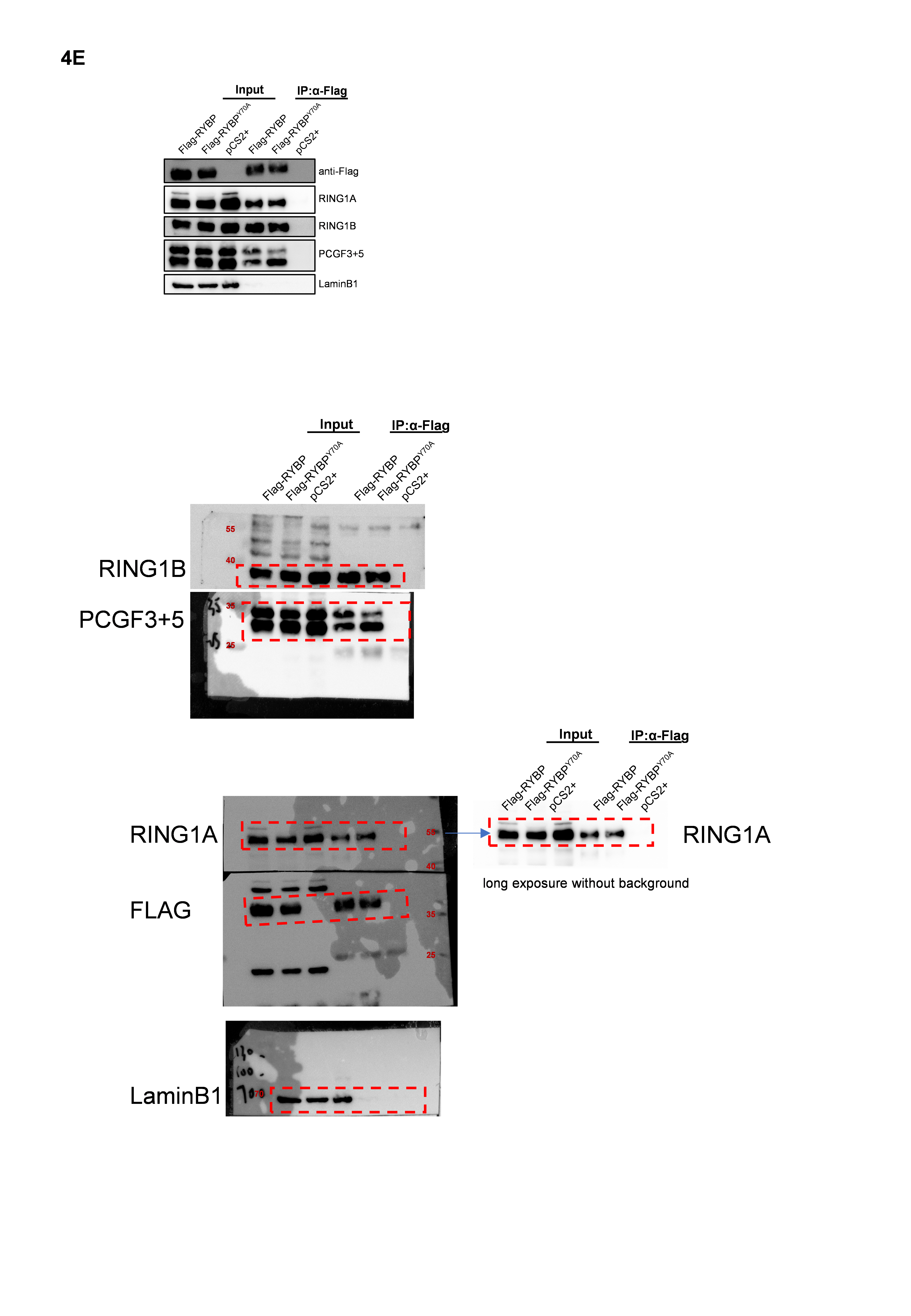

Supplement: Supplementary file 6 — Source data Fig. 4 [file 44318_2026_729_MOESM6_ESM.zip › fig4/4E.tif]

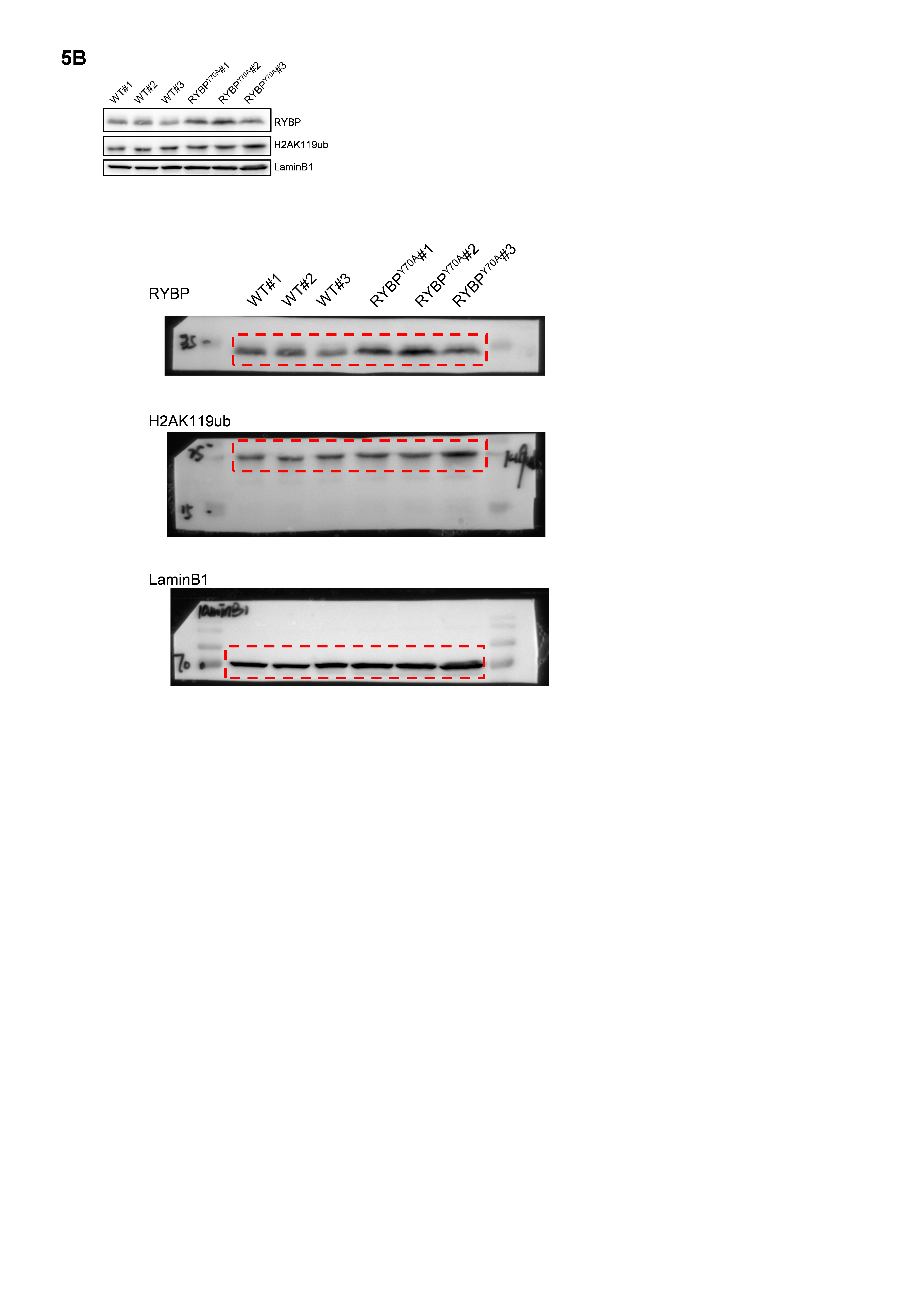

Supplement: Supplementary file 7 — Source data Fig. 5 [file 44318_2026_729_MOESM7_ESM.zip › fig5/5B.tif]

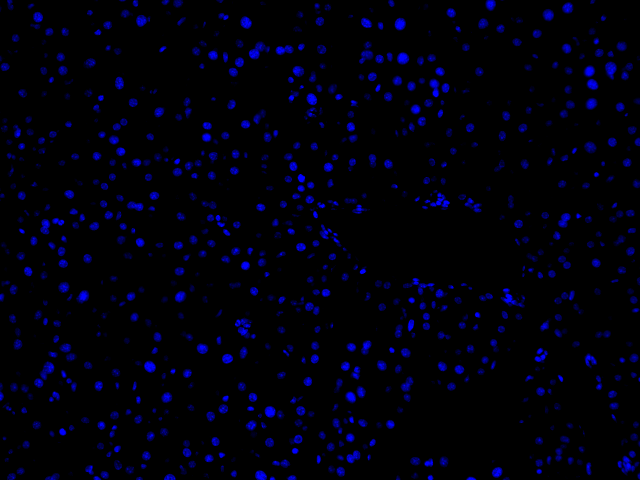

Supplement: Supplementary file 7 — Source data Fig. 5 [file 44318_2026_729_MOESM7_ESM.zip › fig5/Fig5D/Fig5D_WT_Image_CH1(1).tif]

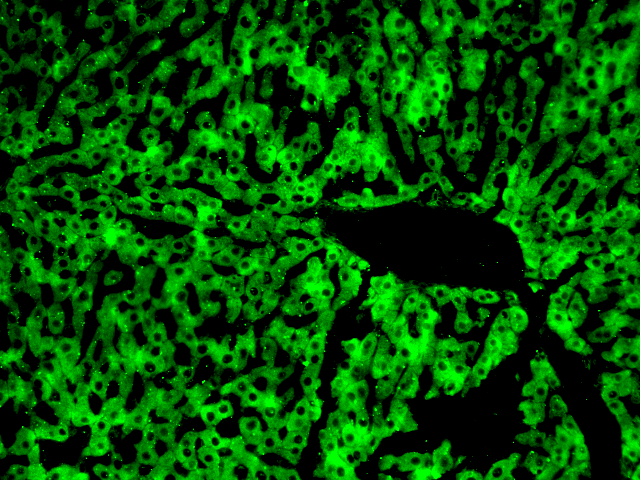

Supplement: Supplementary file 7 — Source data Fig. 5 [file 44318_2026_729_MOESM7_ESM.zip › fig5/Fig5D/Fig5D_WT_Image_CH2(1).tif]

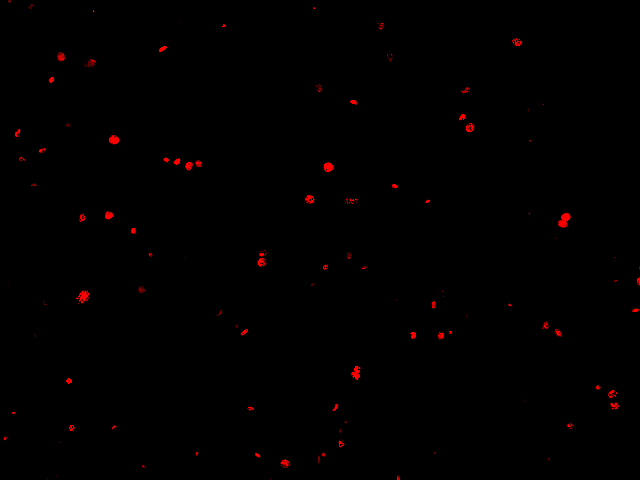

Supplement: Supplementary file 7 — Source data Fig. 5 [file 44318_2026_729_MOESM7_ESM.zip › fig5/Fig5D/Fig5D_WT_Image_CH3(1).tif]

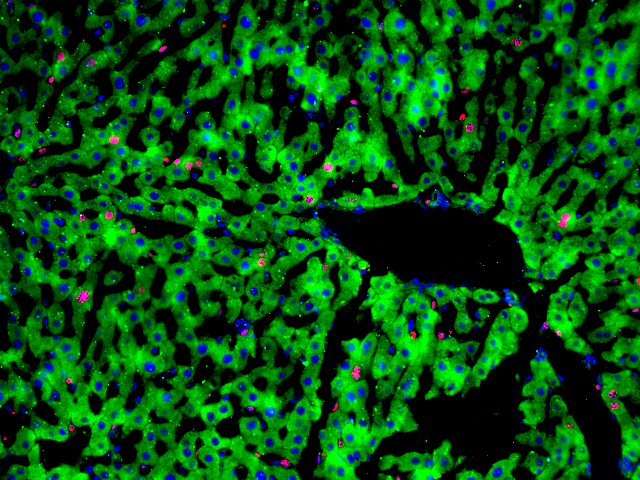

Supplement: Supplementary file 7 — Source data Fig. 5 [file 44318_2026_729_MOESM7_ESM.zip › fig5/Fig5D/Fig5D_WT_Image_Overlay(1).tif]

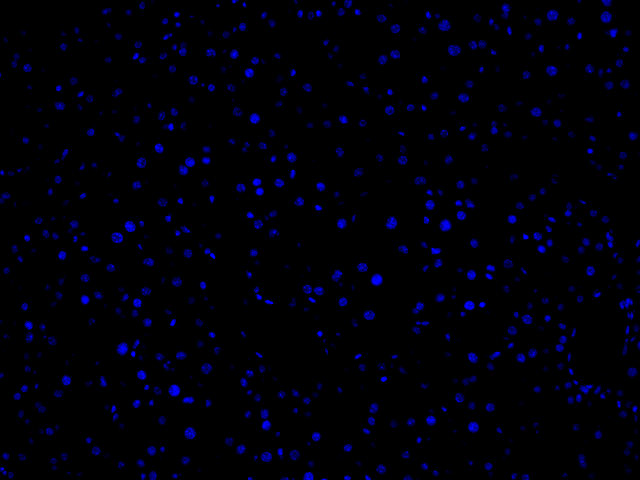

Supplement: Supplementary file 7 — Source data Fig. 5 [file 44318_2026_729_MOESM7_ESM.zip › fig5/Fig5D/Fig5D_Y70A_Image_CH1.tif]

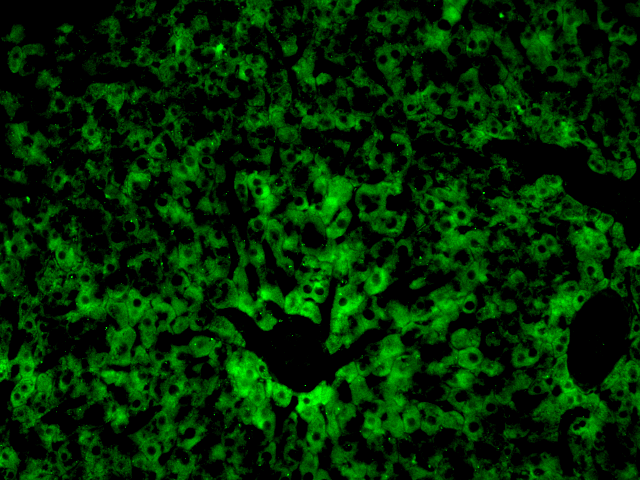

Supplement: Supplementary file 7 — Source data Fig. 5 [file 44318_2026_729_MOESM7_ESM.zip › fig5/Fig5D/Fig5D_Y70A_Image_CH2.tif]

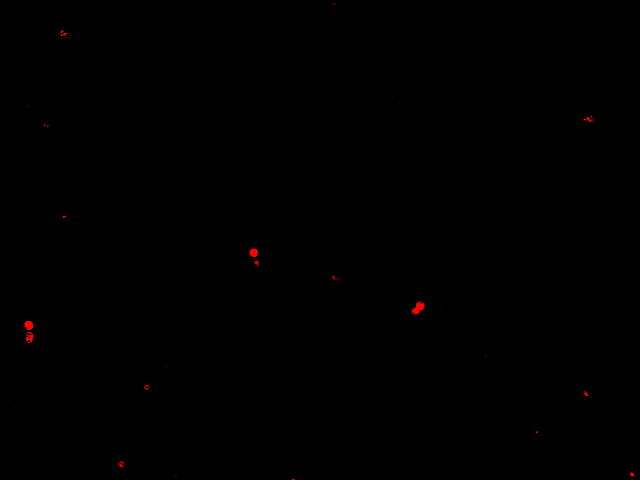

Supplement: Supplementary file 7 — Source data Fig. 5 [file 44318_2026_729_MOESM7_ESM.zip › fig5/Fig5D/Fig5D_Y70A_Image_CH3.tif]

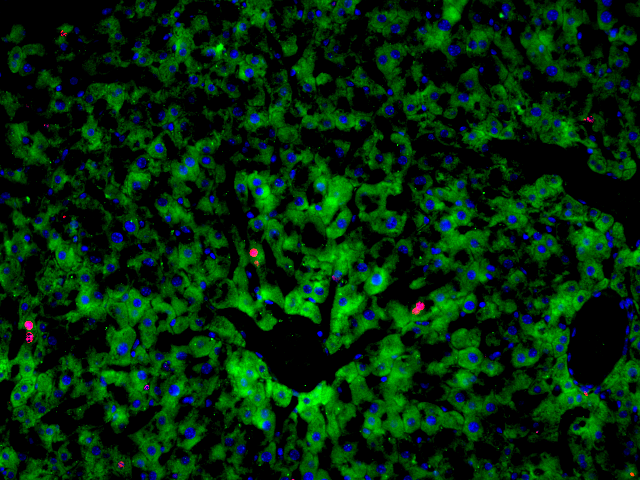

Supplement: Supplementary file 7 — Source data Fig. 5 [file 44318_2026_729_MOESM7_ESM.zip › fig5/Fig5D/Fig5D_Y70A_Image_Overlay.tif]

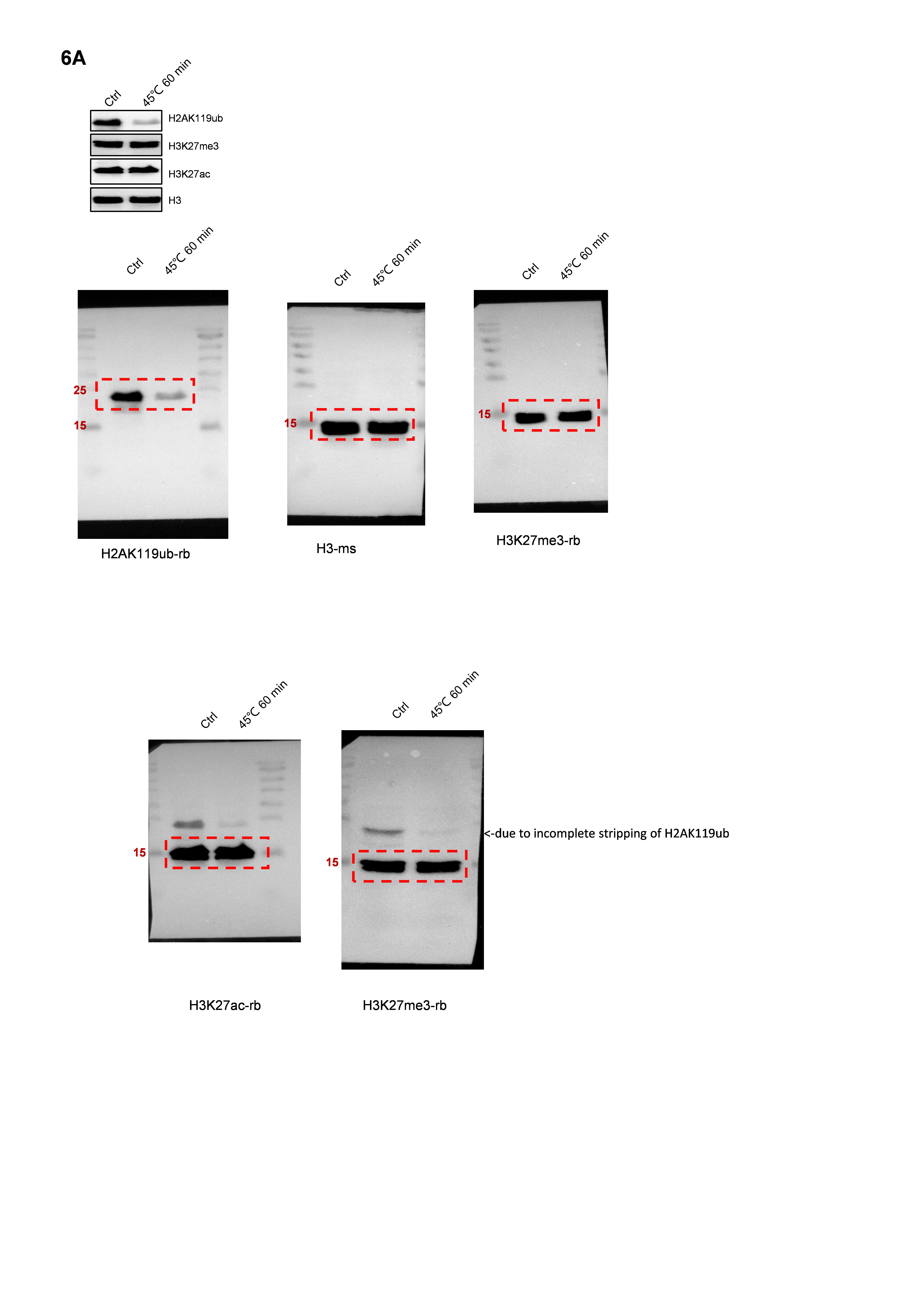

Supplement: Supplementary file 8 — Source data Fig. 6 [file 44318_2026_729_MOESM8_ESM.zip › fig6/6A.tif]

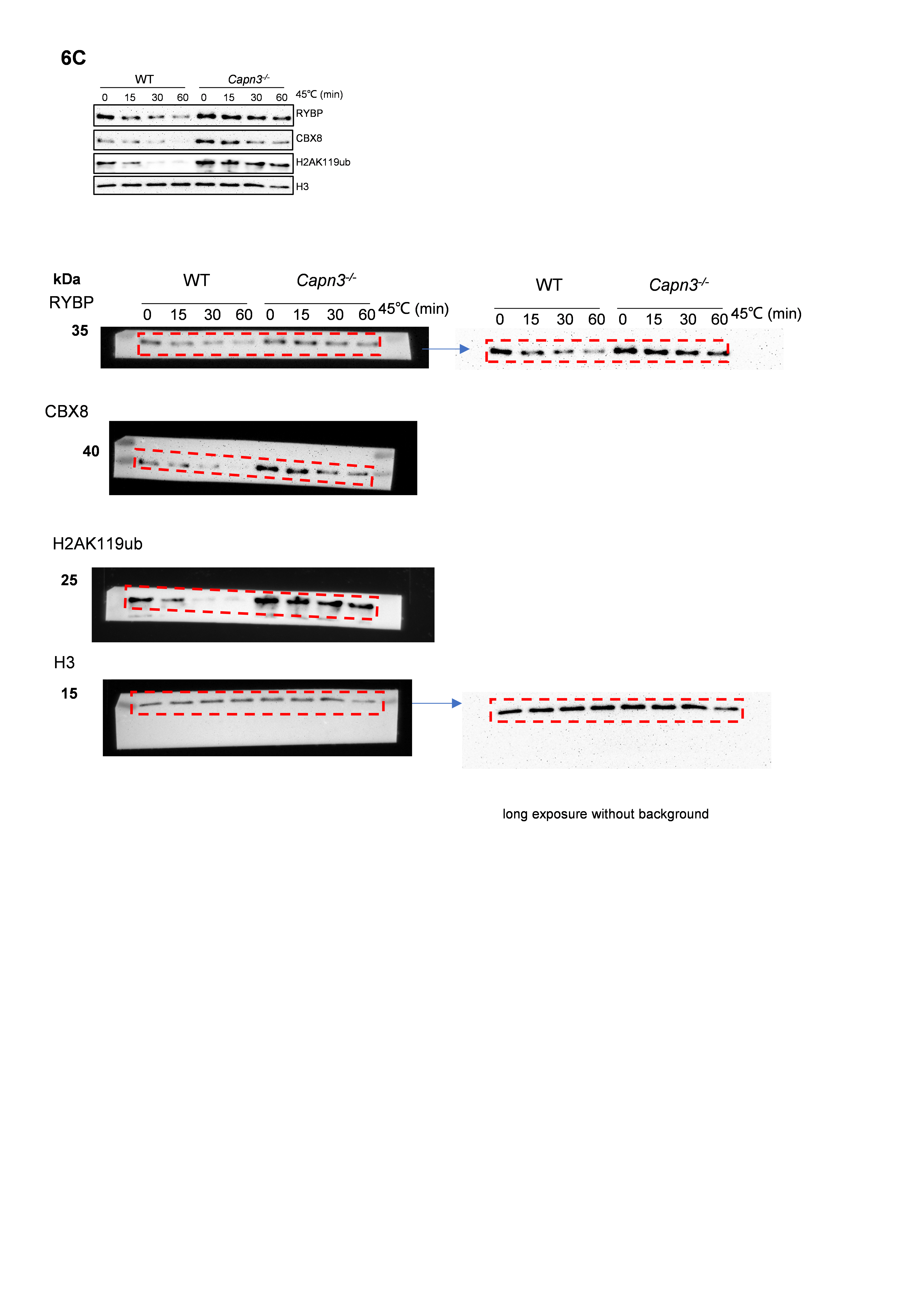

Supplement: Supplementary file 8 — Source data Fig. 6 [file 44318_2026_729_MOESM8_ESM.zip › fig6/6C.tif]

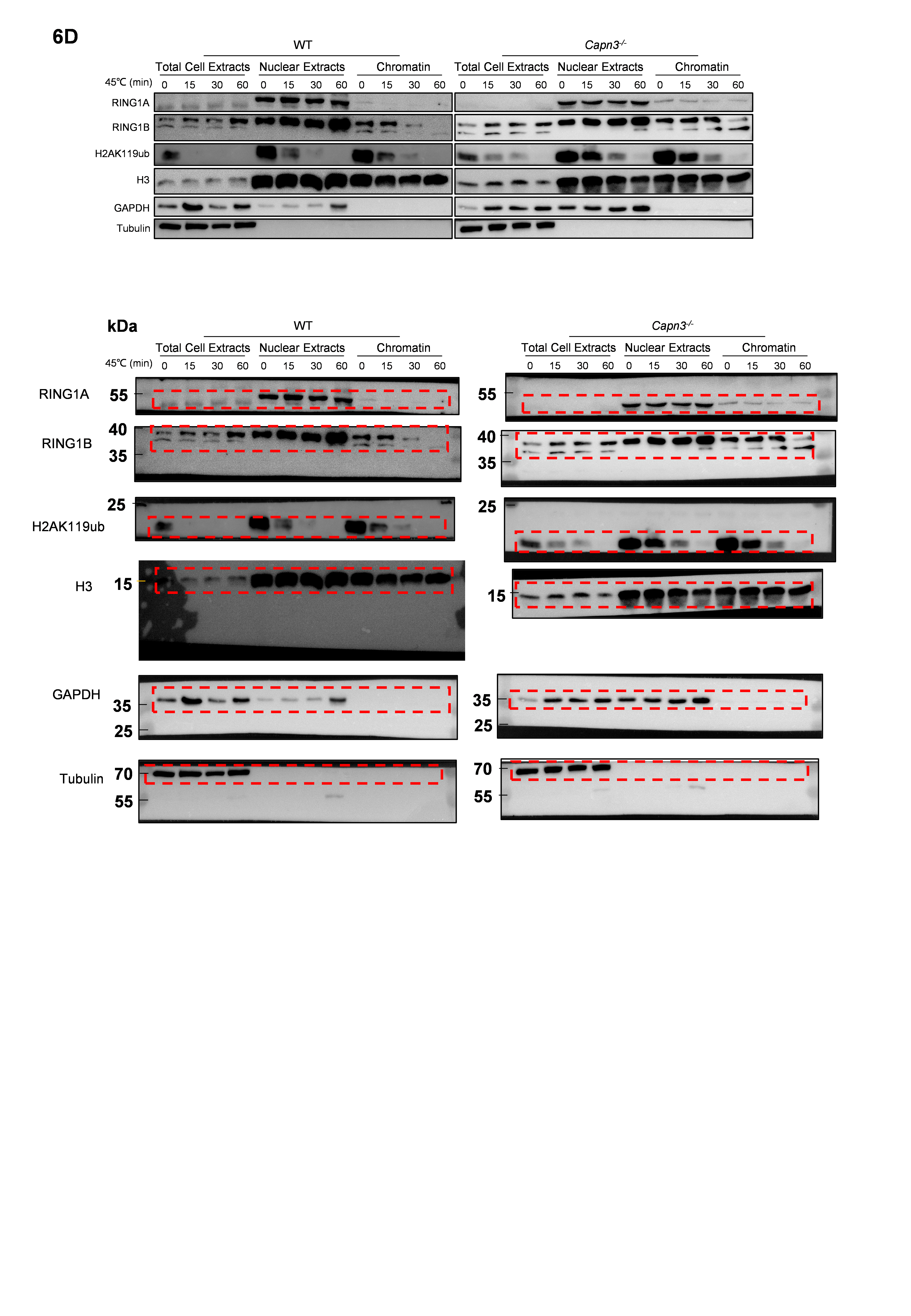

Supplement: Supplementary file 8 — Source data Fig. 6 [file 44318_2026_729_MOESM8_ESM.zip › fig6/6D.tif]

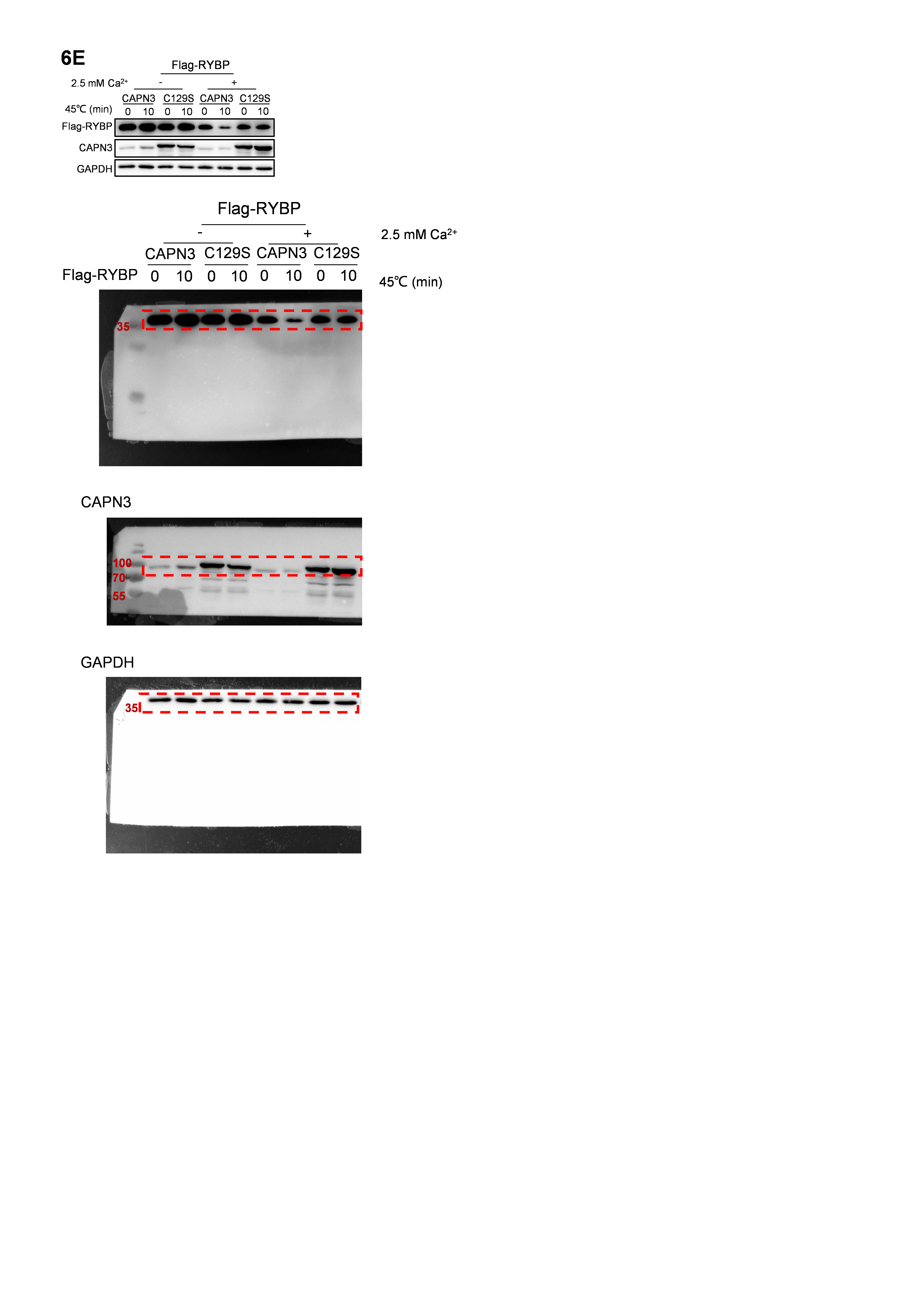

Supplement: Supplementary file 8 — Source data Fig. 6 [file 44318_2026_729_MOESM8_ESM.zip › fig6/6E.tif]

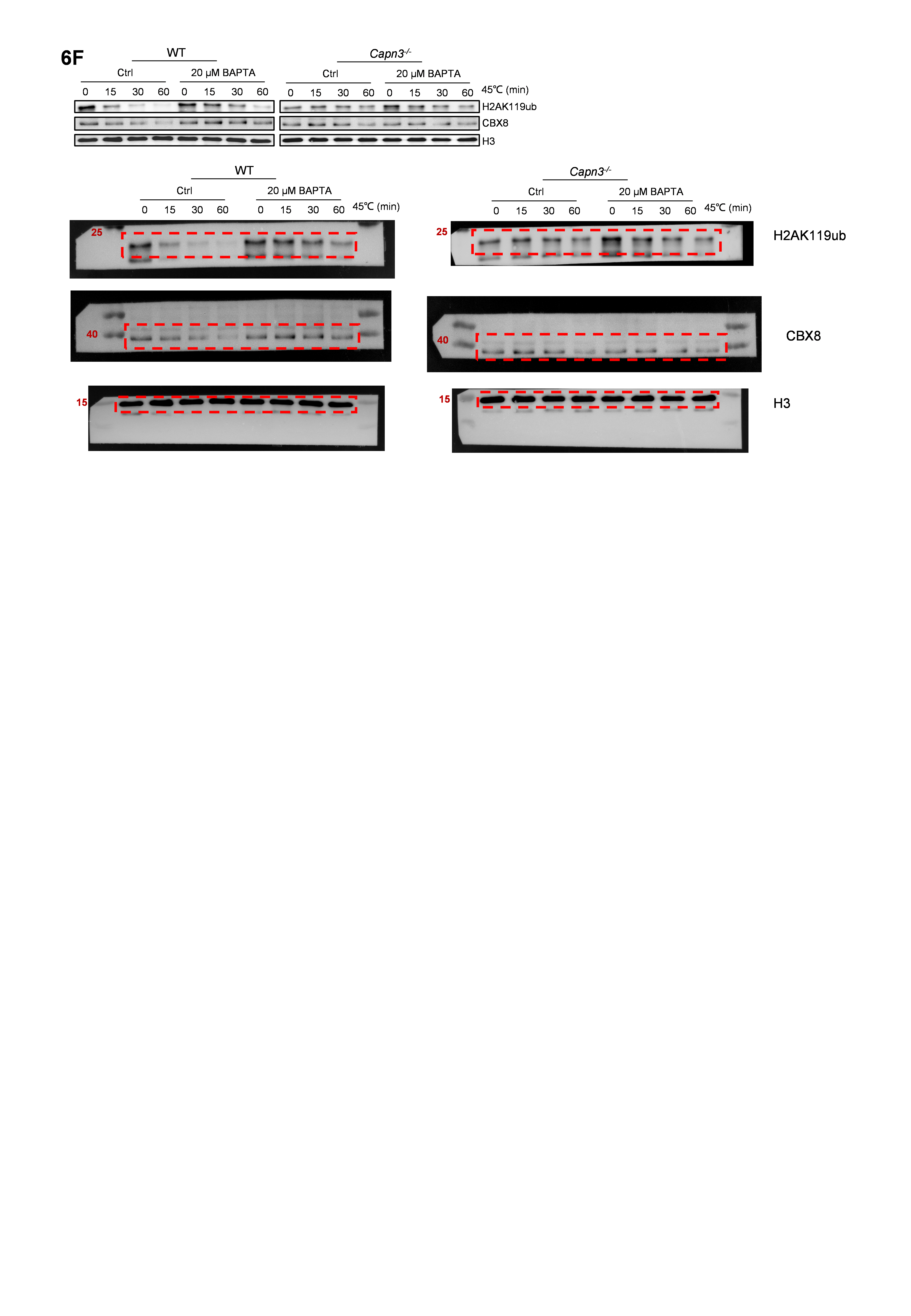

Supplement: Supplementary file 8 — Source data Fig. 6 [file 44318_2026_729_MOESM8_ESM.zip › fig6/6F.tif]

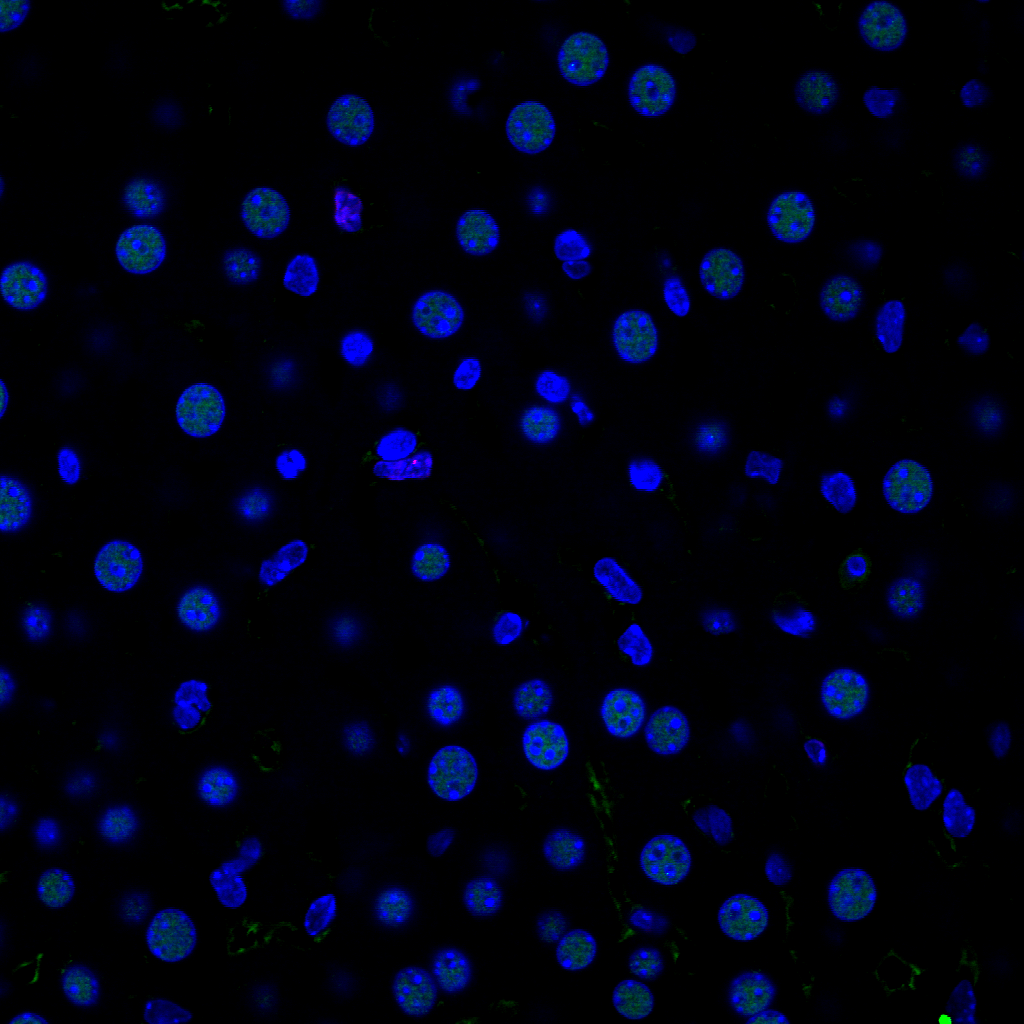

Supplement: Supplementary file 9 — Figure EV2B Source Data [file 44318_2026_729_MOESM9_ESM.zip › figEV2B/CAPN3--_0h/KO2_0h_6_.tif]

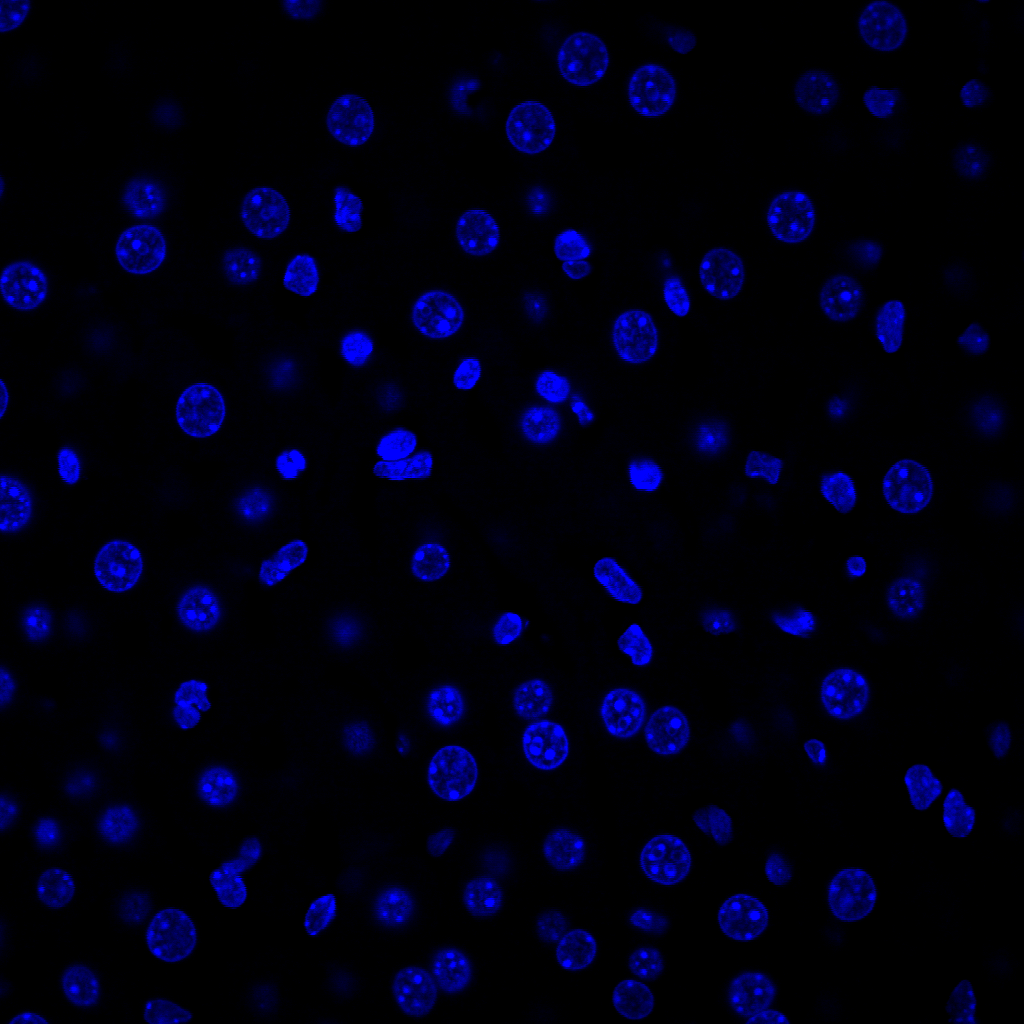

Supplement: Supplementary file 9 — Figure EV2B Source Data [file 44318_2026_729_MOESM9_ESM.zip › figEV2B/CAPN3--_0h/KO2_0h_6_C001.tif]

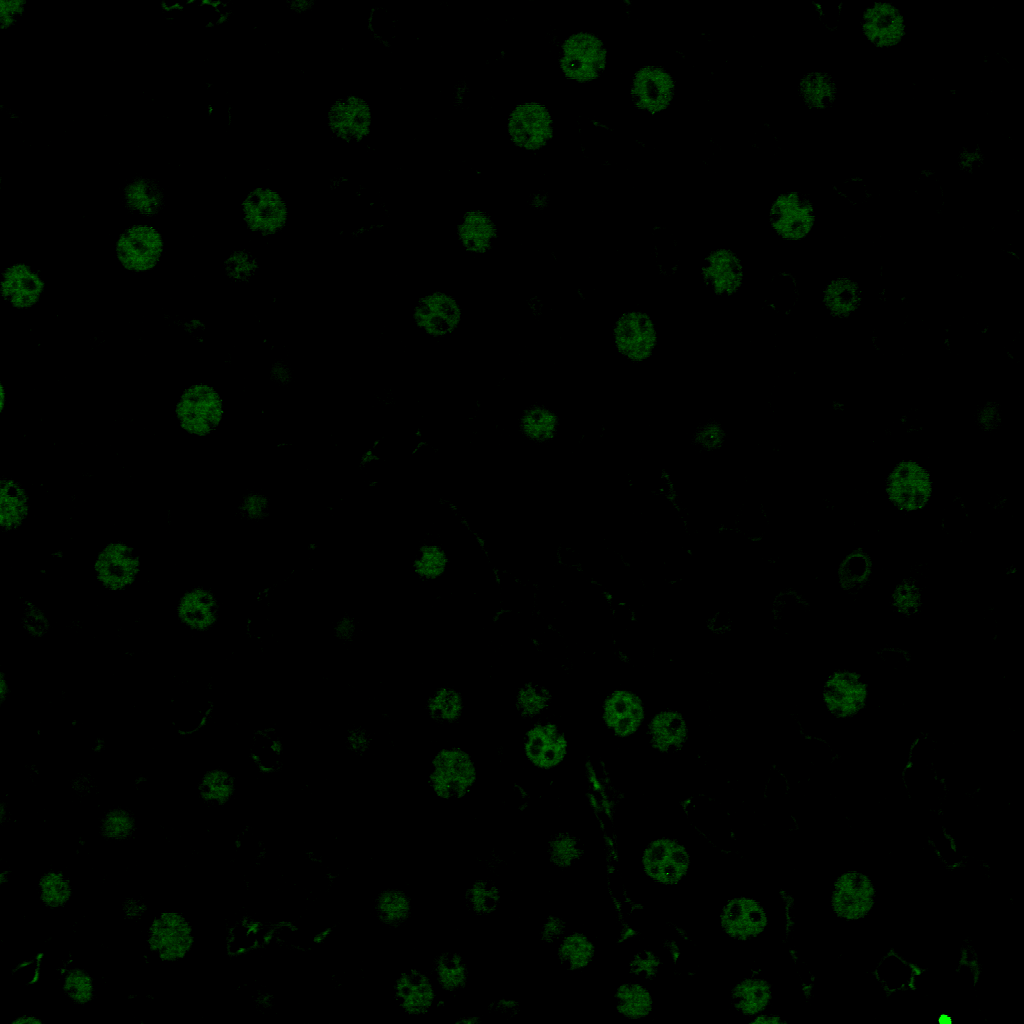

Supplement: Supplementary file 9 — Figure EV2B Source Data [file 44318_2026_729_MOESM9_ESM.zip › figEV2B/CAPN3--_0h/KO2_0h_6_C002.tif]

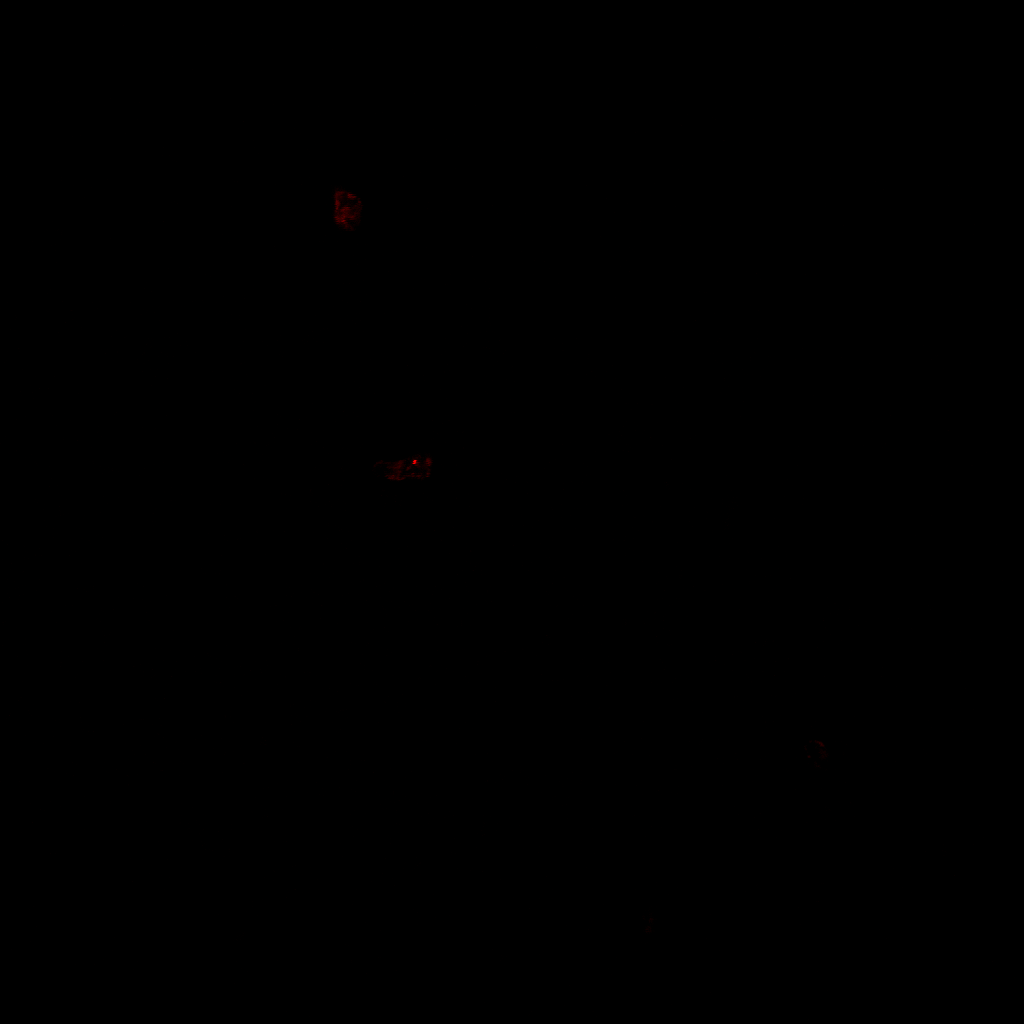

Supplement: Supplementary file 9 — Figure EV2B Source Data [file 44318_2026_729_MOESM9_ESM.zip › figEV2B/CAPN3--_0h/KO2_0h_6_C003.tif]

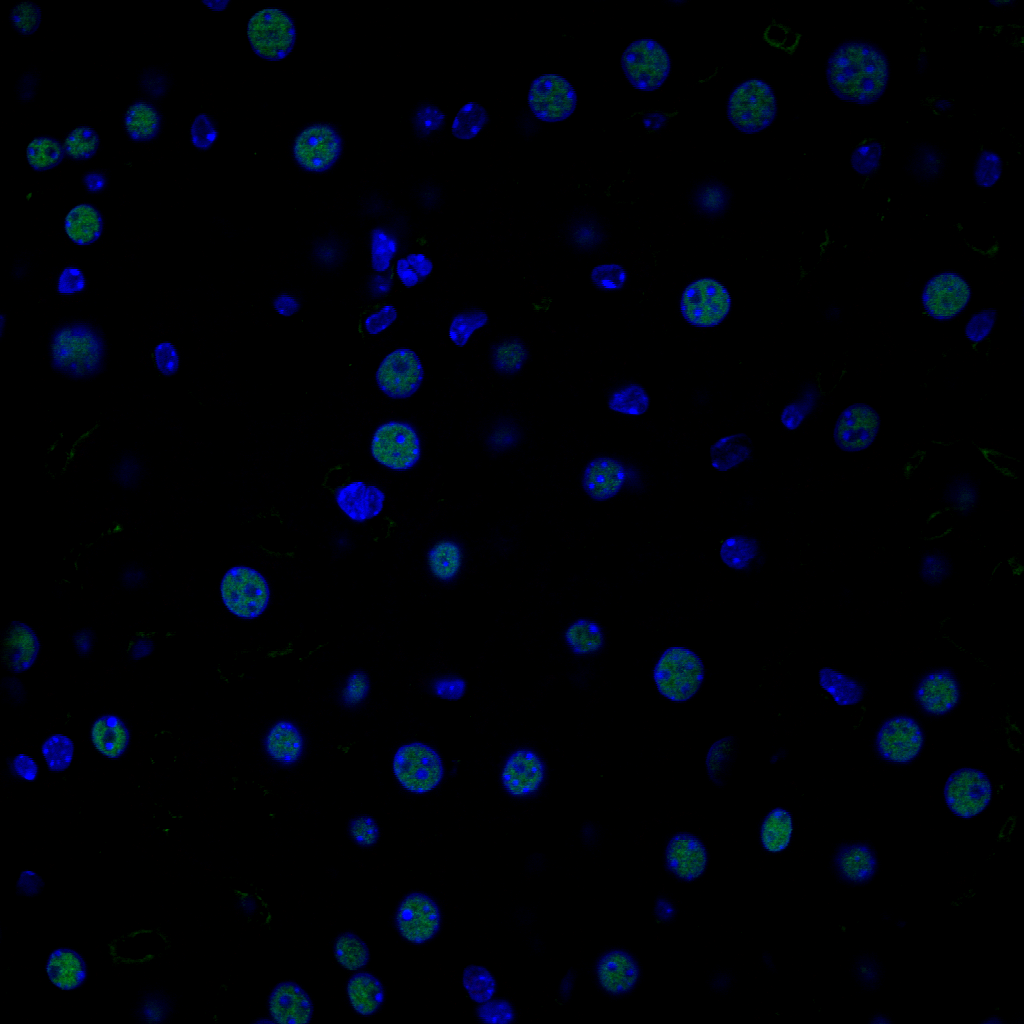

Supplement: Supplementary file 9 — Figure EV2B Source Data [file 44318_2026_729_MOESM9_ESM.zip › figEV2B/CAPN3--_16h/KO2_16h_2_.tif]

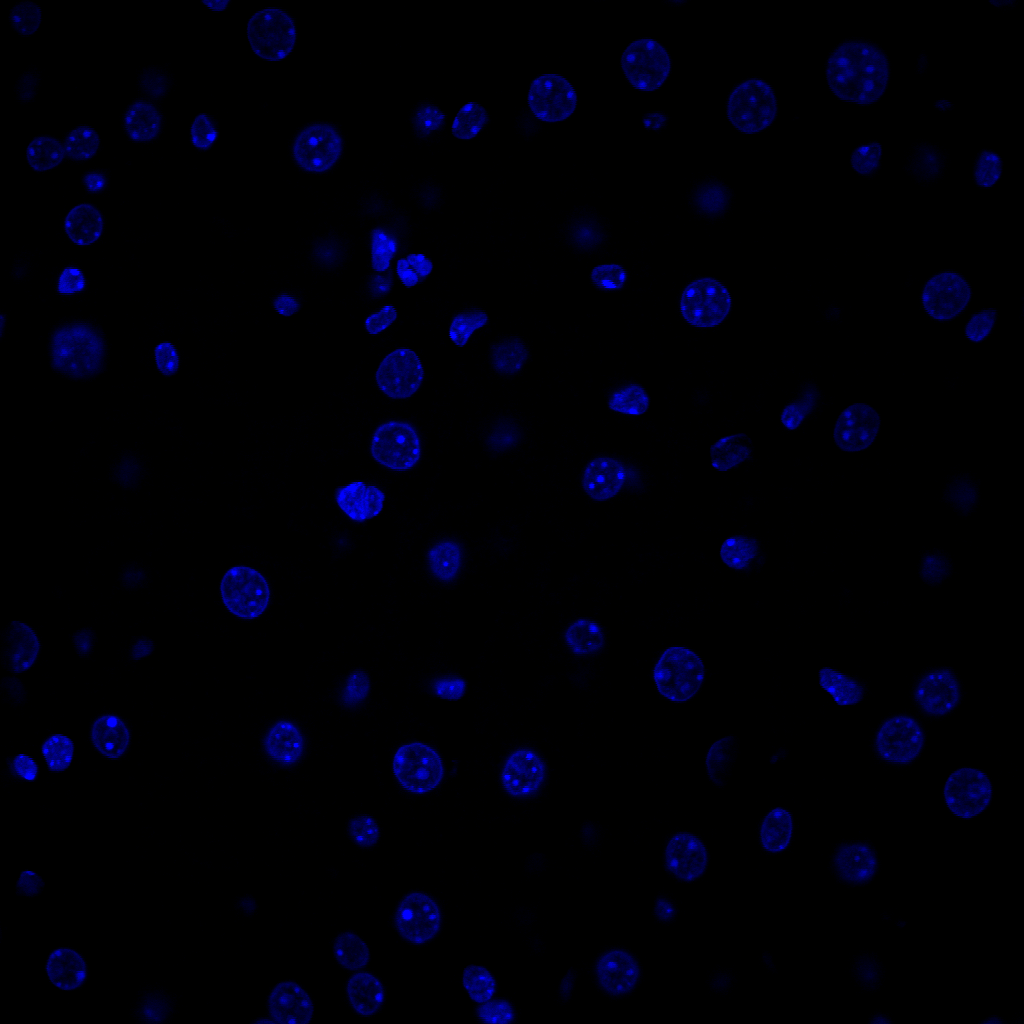

Supplement: Supplementary file 9 — Figure EV2B Source Data [file 44318_2026_729_MOESM9_ESM.zip › figEV2B/CAPN3--_16h/KO2_16h_2_C001.tif]

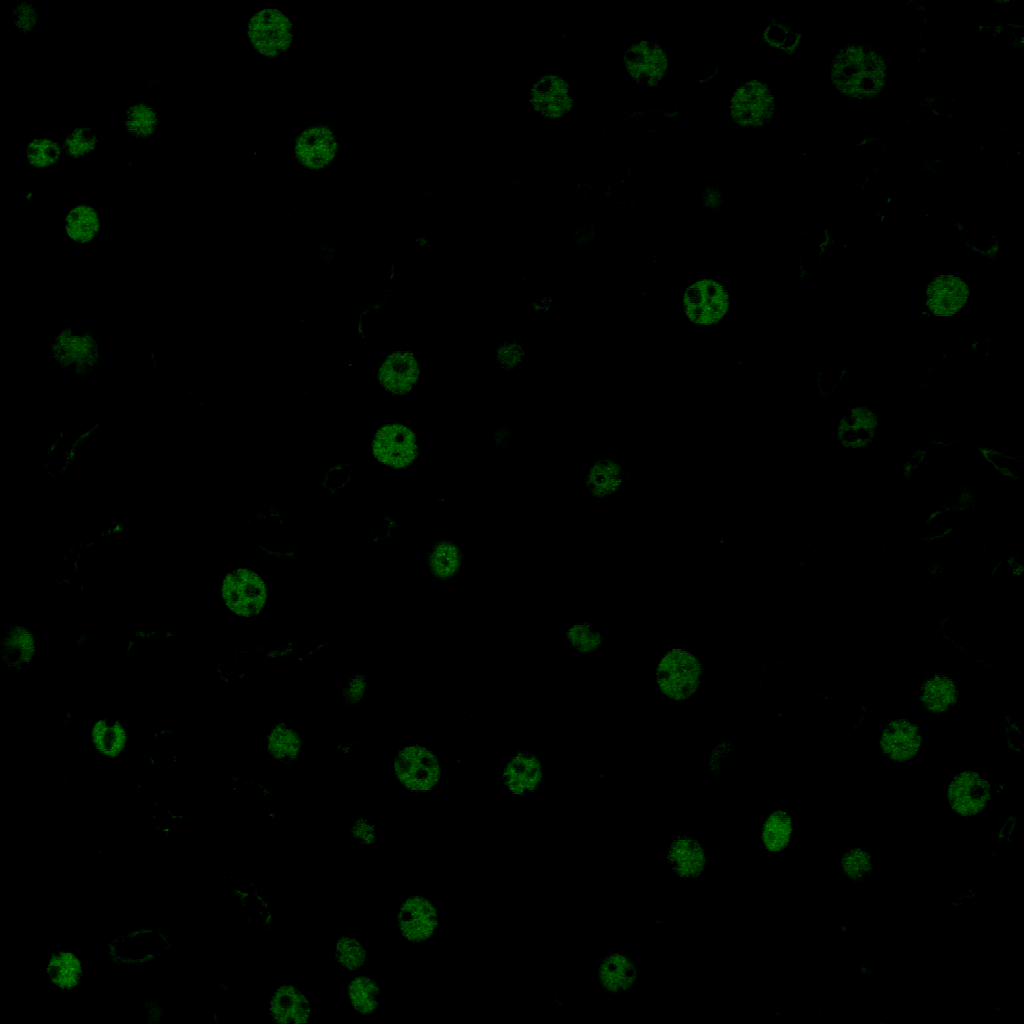

Supplement: Supplementary file 9 — Figure EV2B Source Data [file 44318_2026_729_MOESM9_ESM.zip › figEV2B/CAPN3--_16h/KO2_16h_2_C002.tif]

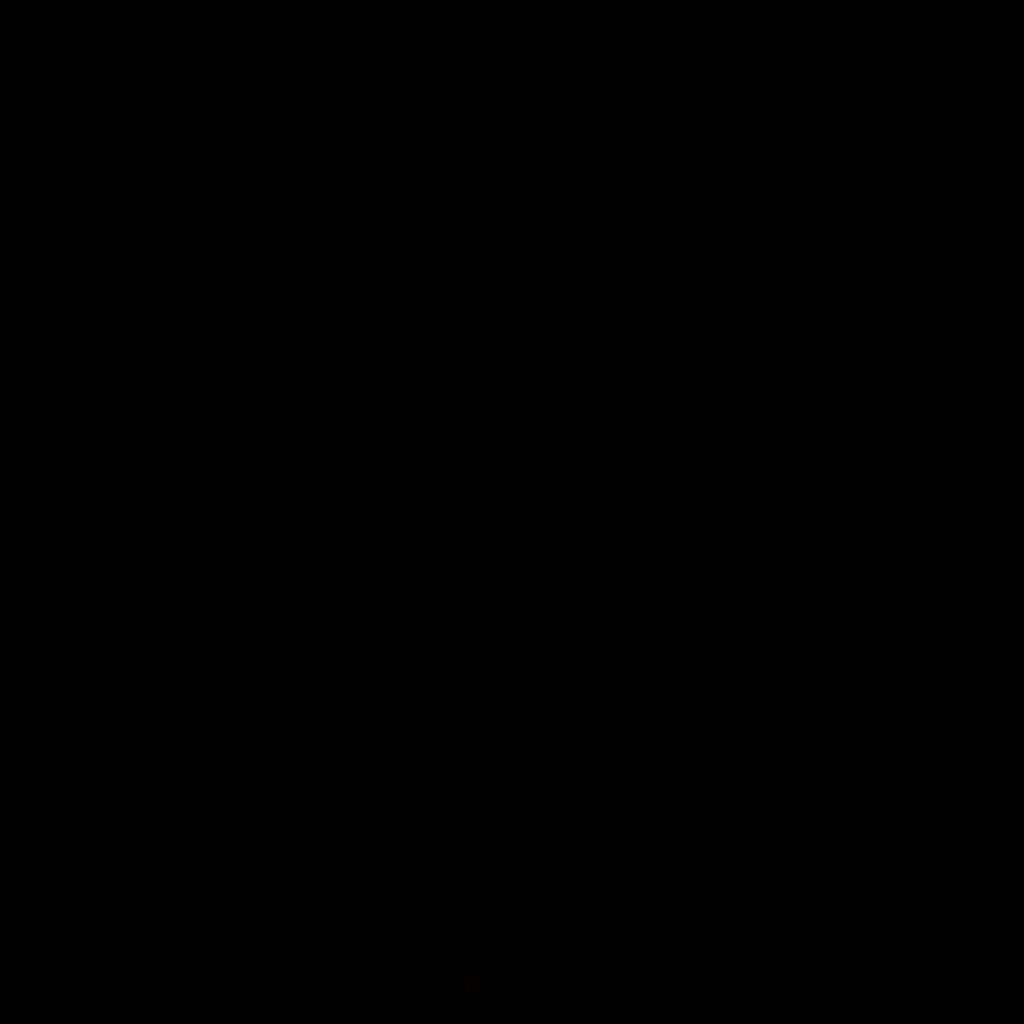

Supplement: Supplementary file 9 — Figure EV2B Source Data [file 44318_2026_729_MOESM9_ESM.zip › figEV2B/CAPN3--_16h/KO2_16h_2_C003.tif]

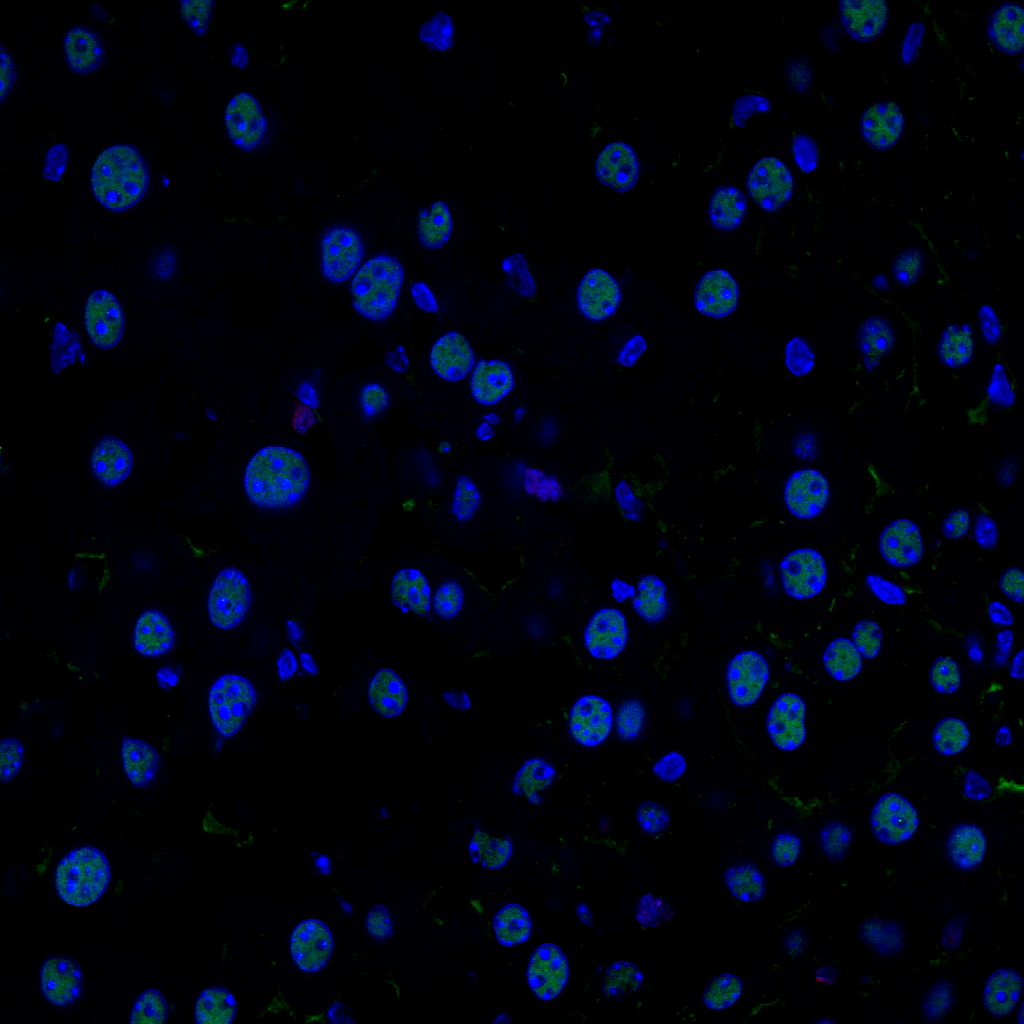

Supplement: Supplementary file 9 — Figure EV2B Source Data [file 44318_2026_729_MOESM9_ESM.zip › figEV2B/WT_0h/WT3_0h_6_.tif]

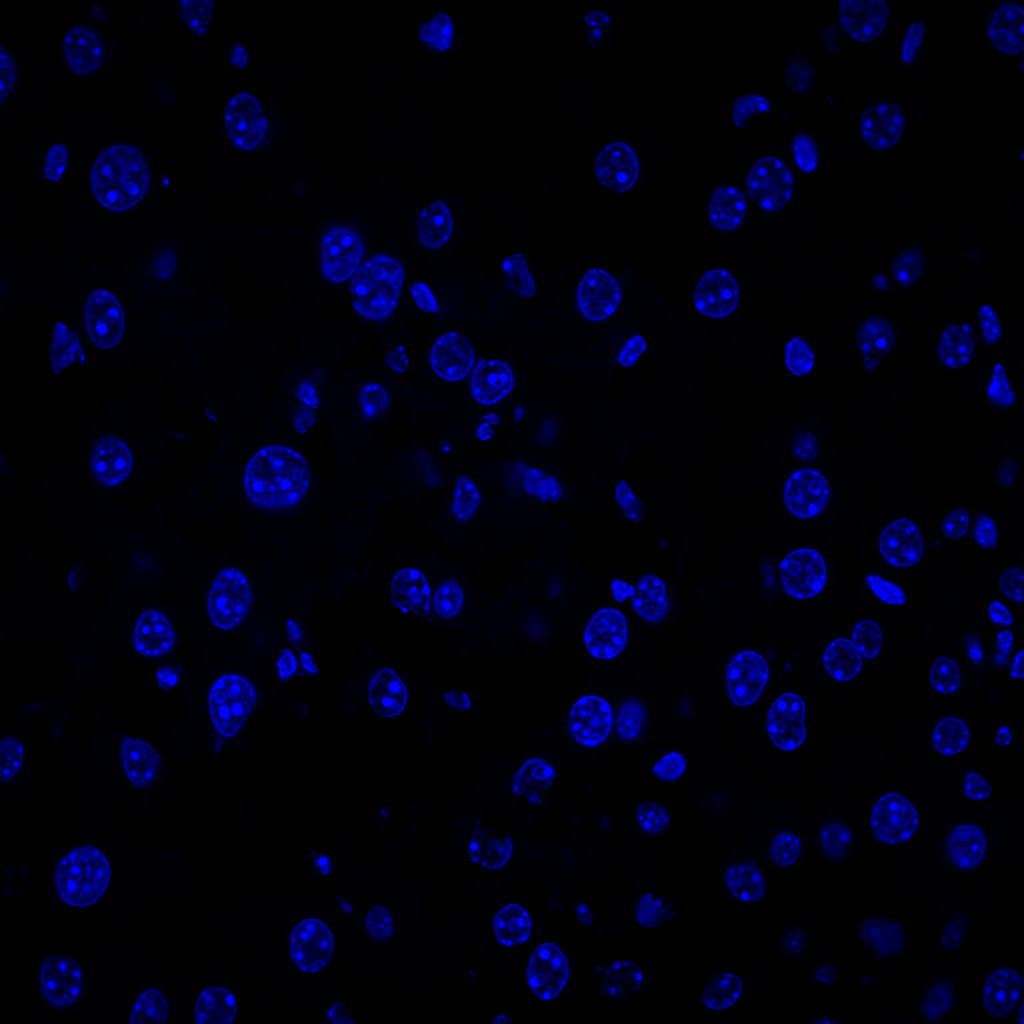

Supplement: Supplementary file 9 — Figure EV2B Source Data [file 44318_2026_729_MOESM9_ESM.zip › figEV2B/WT_0h/WT3_0h_6_C001.tif]

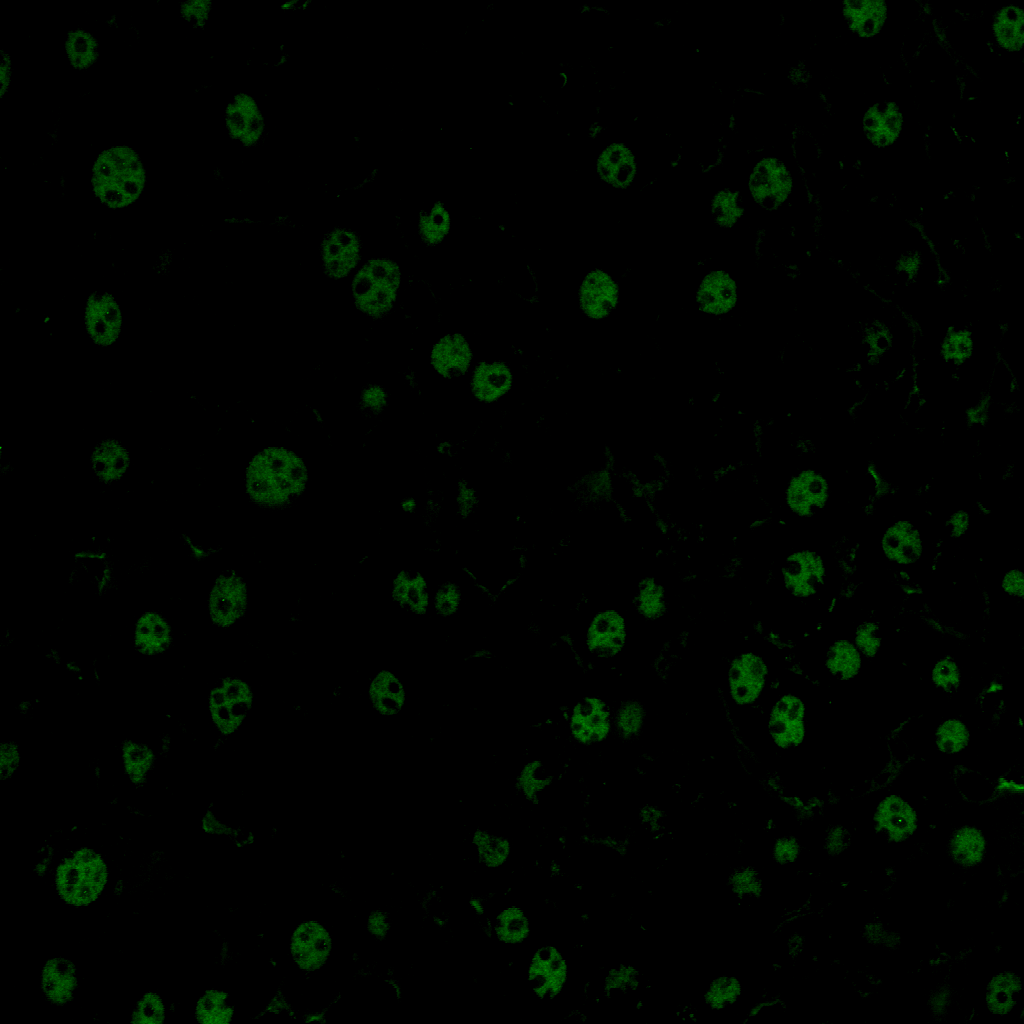

Supplement: Supplementary file 9 — Figure EV2B Source Data [file 44318_2026_729_MOESM9_ESM.zip › figEV2B/WT_0h/WT3_0h_6_C002.tif]

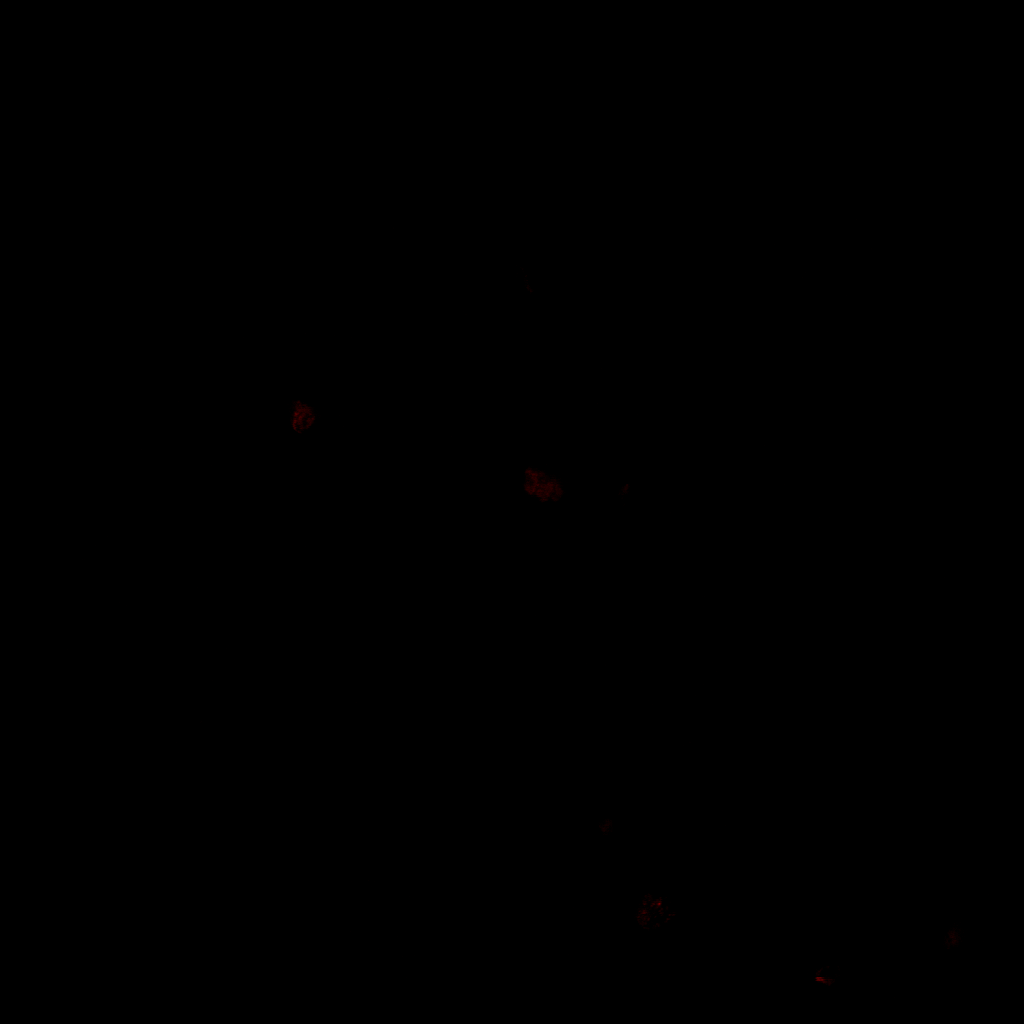

Supplement: Supplementary file 9 — Figure EV2B Source Data [file 44318_2026_729_MOESM9_ESM.zip › figEV2B/WT_0h/WT3_0h_6_C003.tif]

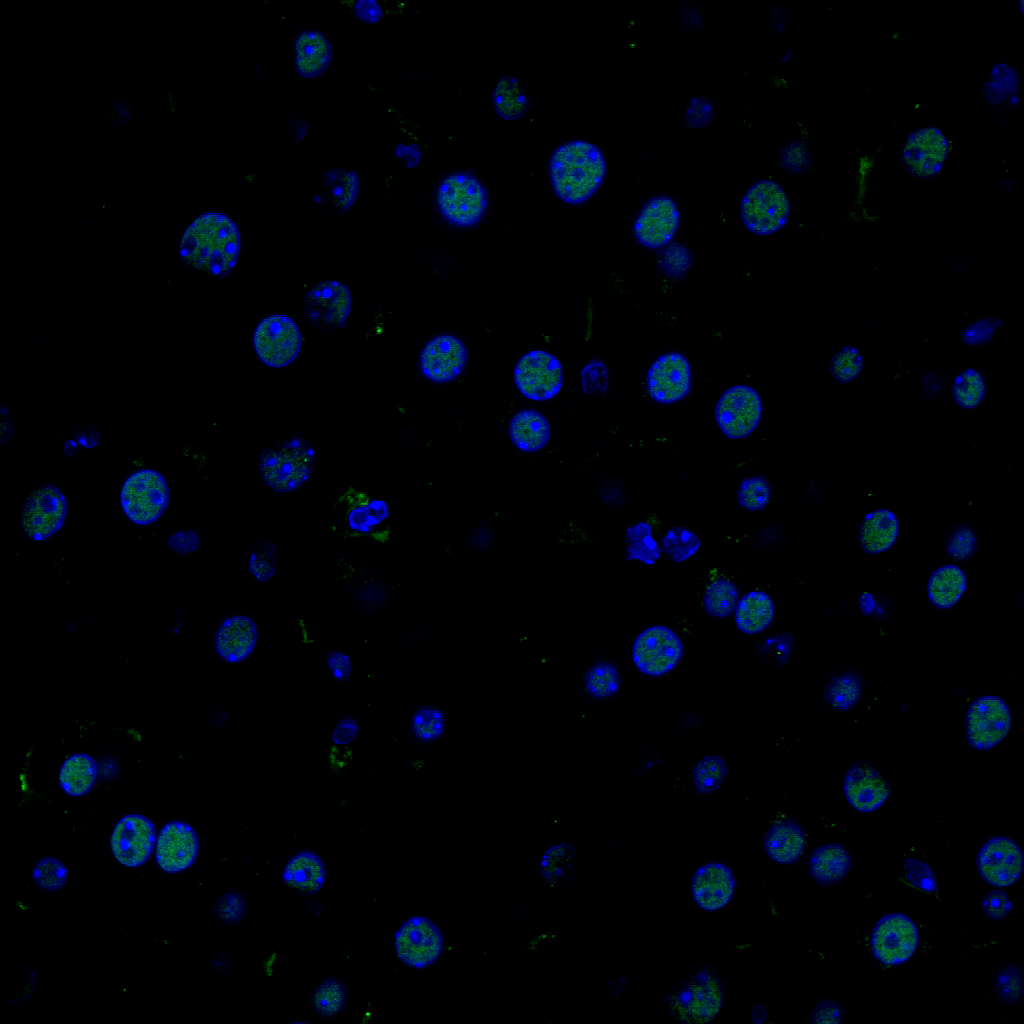

Supplement: Supplementary file 9 — Figure EV2B Source Data [file 44318_2026_729_MOESM9_ESM.zip › figEV2B/WT_16h/WT2_16h_3_.tif]

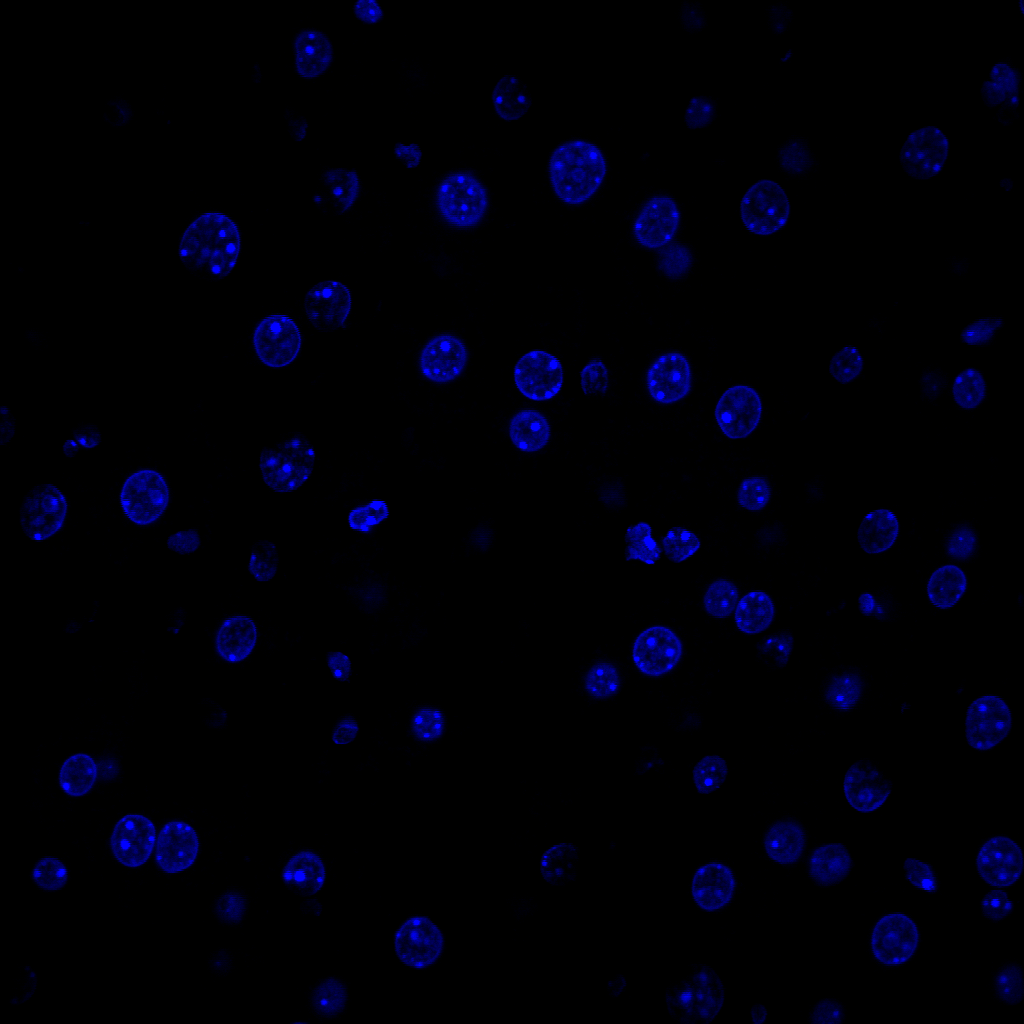

Supplement: Supplementary file 9 — Figure EV2B Source Data [file 44318_2026_729_MOESM9_ESM.zip › figEV2B/WT_16h/WT2_16h_3_C001.tif]

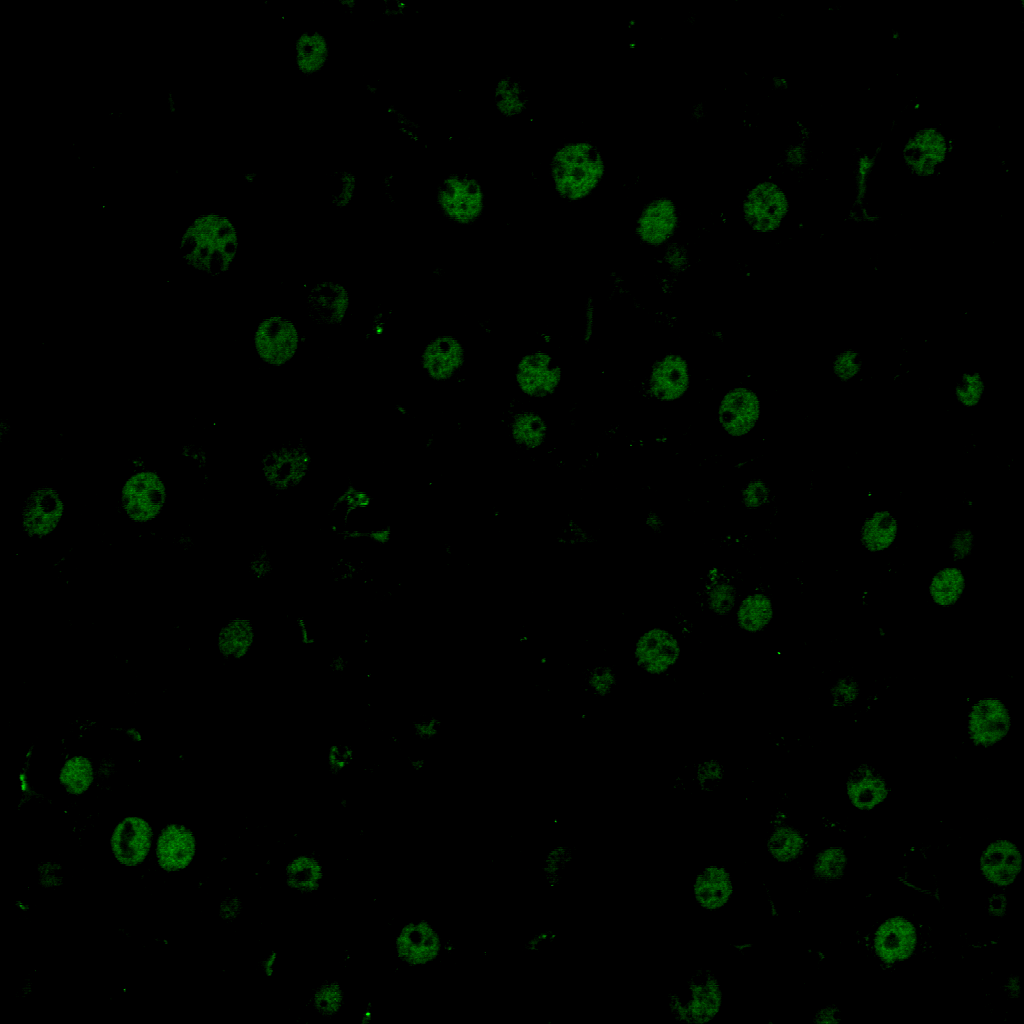

Supplement: Supplementary file 9 — Figure EV2B Source Data [file 44318_2026_729_MOESM9_ESM.zip › figEV2B/WT_16h/WT2_16h_3_C002.tif]

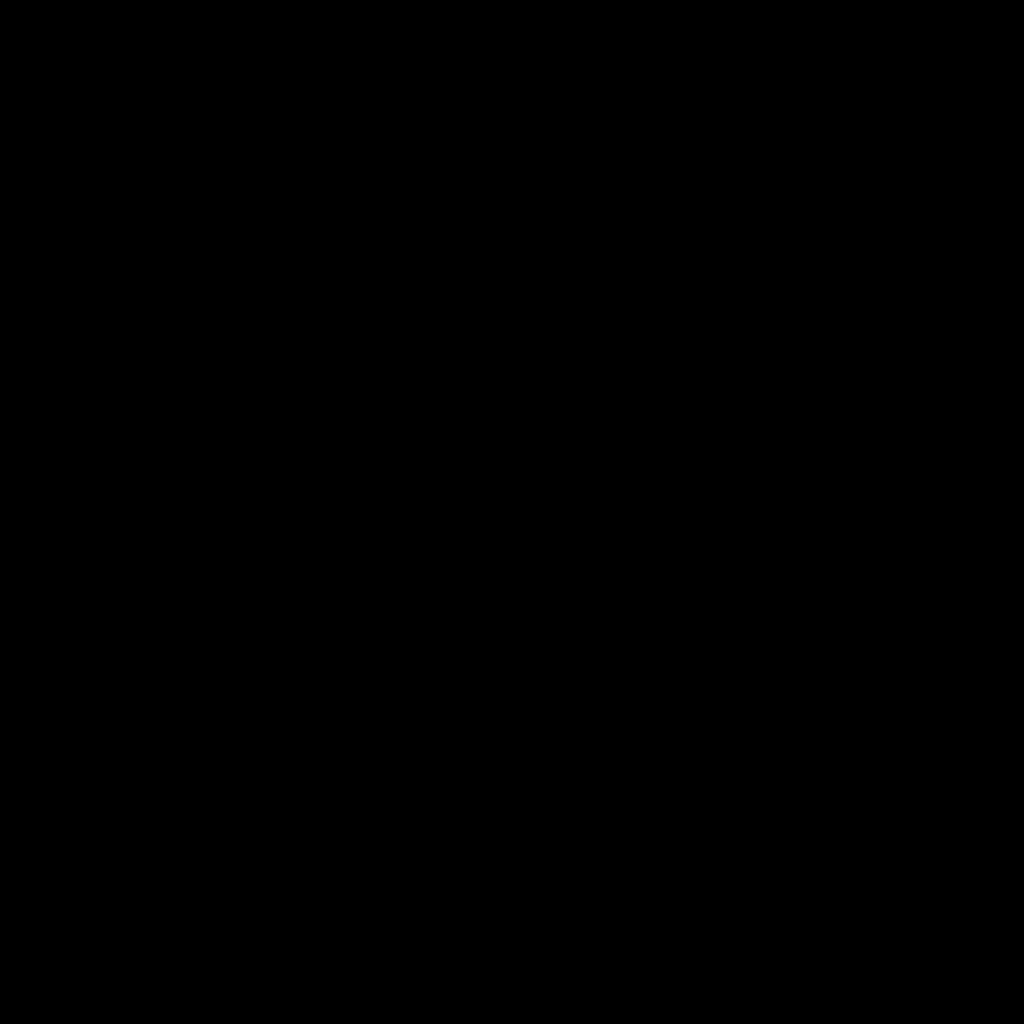

Supplement: Supplementary file 9 — Figure EV2B Source Data [file 44318_2026_729_MOESM9_ESM.zip › figEV2B/WT_16h/WT2_16h_3_C003.tif]
