# Supplementary material for: Protease-mediated PRC1 dissociation promotes H2AK119ub remodeling during stress responses
Source: EMBO J. 2026 Mar 11;45(8):2561–86. doi: 10.1038/s44318-026-00729-9 (PMC13083880; doi:10.1038/s44318-026-00729-9)
Supplement: Supplementary file 10 — Expanded View Figures [file 44318_2026_729_MOESM10_ESM.pdf]

Expanded View Figures

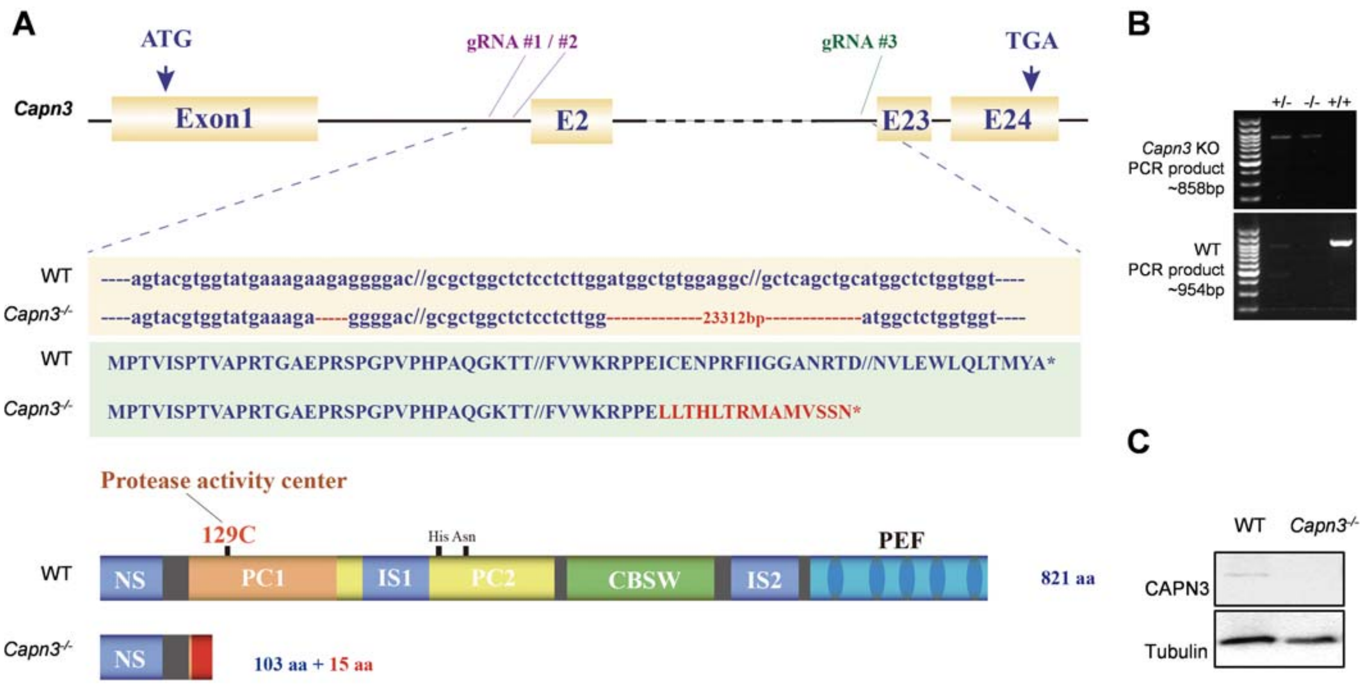

Figure EV1. Generation of *Capn3* KO mice by CRISPR/Cas9 genome editing system.

(**A**) Scheme of gRNA target sites for generating *Capn3* KO mice and the alignments of nucleotide sequences (top), protein sequence (middle), and domain (bottom) from wild-type (WT) and *Capn3*$^{-/-}$ mice. (**B**) *Capn3*$^{-/-}$ confirmed by PCR genotyping. The first pair of primers can only detect the KO product about 858 bp in *Capn3*$^{+/-}$ and *Capn3*$^{-/-}$ (top), the second pair of primers can only detect the WT product about 954 bp in *Capn3*$^{+/-}$ and WT (bottom). (**C**) Western blot analysis of CAPN3 protein in WT and *Capn3*$^{-/-}$ hepatocytes, which were isolated by perfusion from the WT and *Capn3*$^{-/-}$ mice livers.

 © The Author(s)

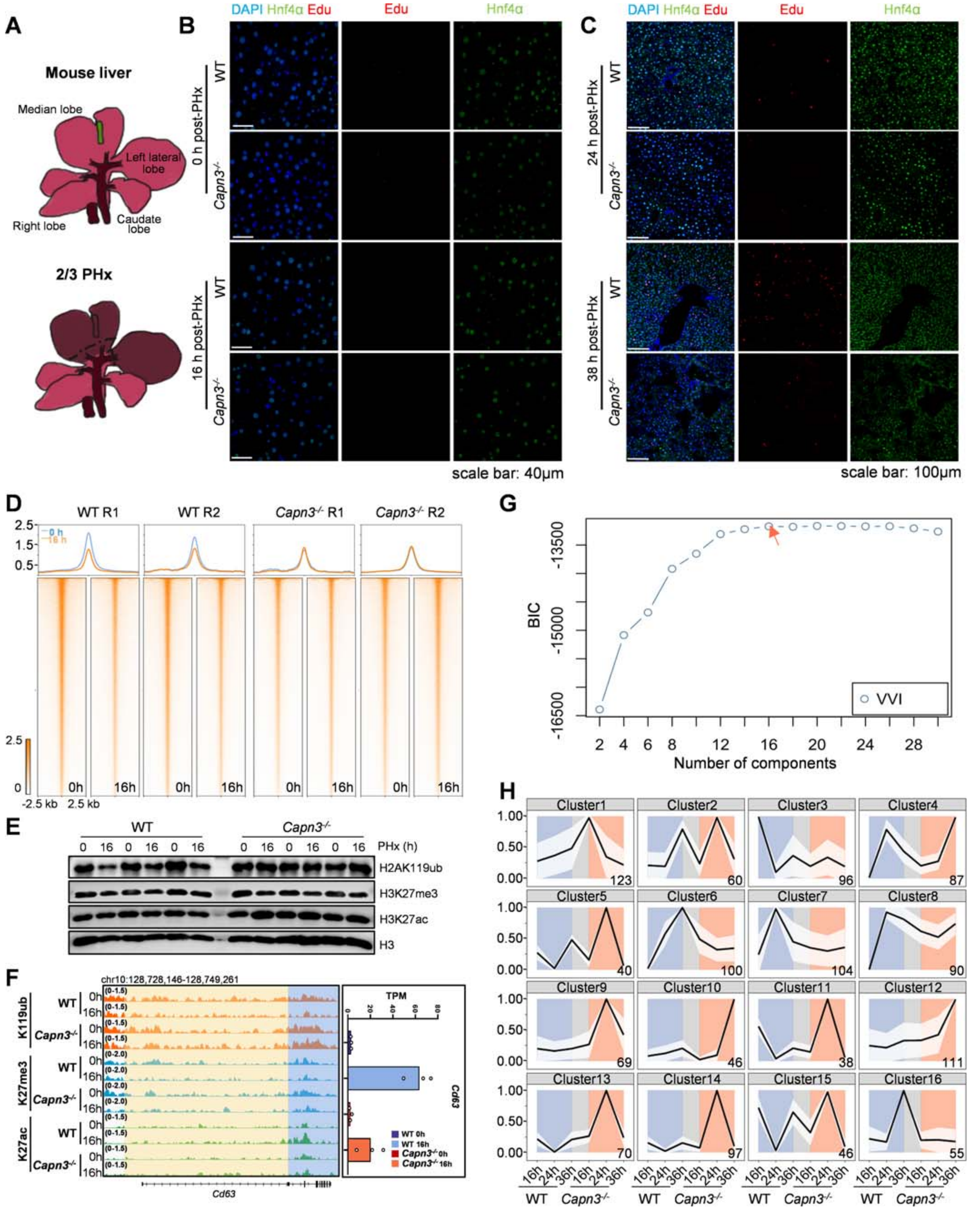
A
Mouse liver
Median lobe
Left lateral lobe
Right lobe
Caudate lobe
2/3 PHx
B
DAPI Hnf4α Edu
Edu
Hnf4α
0 h post-PHx
16 h post-PHx
WT
Capn3-/-
scale bar: 40μm
C
24 h post-PHx
38 h post-PHx
scale bar: 100μm
D
WT R1
WT R2
Capn3-/- R1
Capn3-/- R2
0 h
16 h
-2.5 kb 2.5 kb
E
PHx (h)
H2AK119ub
H3K27me3
H3K27ac
H3
F
chr10:128,728,146-128,749,261
K119ub
K27me3
K27ac
TPM
Cd63
WT 0h
WT 16h
Capn3-/- 0h
Capn3-/- 16h
G
BIC
VVI
Number of components
H
Cluster1
Cluster2
Cluster3
Cluster4
Cluster5
Cluster6
Cluster7
Cluster8
Cluster9
Cluster10
Cluster11
Cluster12
Cluster13
Cluster14
Cluster15
Cluster16

Figure EV2. Depletion of CAPN3 delays hepatocyte proliferation and inhibits H2AK119ub remodeling after PHx.

(**A**) Scheme of mouse liver anatomy and positioning of silk threads for knots (dash lines). (**B**) Immunostaining of EdU and Hnf4a (a hepatocyte marker) at 0 h and 16 h post-PHx. Scale bars, 40 μm. (**C**) Immunostaining of EdU and Hnf4a at 24 h and 38 h post-PHx. Scale bars, 100 μm. (**D**) Two independent biological replicates of CUT&Tag profiles and heatmaps of H2AK119ub in WT and *Capn3*$^{-/-}$ hepatocytes at 0 h and 16 h post-PHx at called peaks. Color intensity for each strand represents counts per million (CPM). (**E**) Chromatin fractions were isolated from the livers of three independent WT and *Capn3*$^{-/-}$ mouse. Western blot analysis was performed with the indicated antibodies. (**F**) Genome browser snapshots (left) of H2AK119ub, H3K27me3, and H3K27ac tracks at the *Cd63* loci in WT and *Capn3*$^{-/-}$ hepatocytes at 0 h or 16 h post-PHx. The expression levels (right) of *Cd63* in TPM at 0 h or 16 h post-PHx are summarized in bar charts. The shaded areas highlight the #41_44 (blue) and #42_44 (yellow) regions. (**G**) Bayesian information content (BIC) was computed to assess 2–30 component models to get the optimal cluster number (indicated by orange arrow). (**H**) Covariance clustering of genes near #41_44, #42_44 and #43_44 loci that display a delayed reduction in H2AK119ub (TPM > 0.1 in ≥1 condition, $n = 1232$) across 16, 24, and 36 h PHx in WT and *Capn3*$^{-/-}$ hepatocytes into 16 clusters. Gene counts for each cluster are shown at the bottom right of each corresponding panel. Source data are available online for this figure.

 © The Author(s)

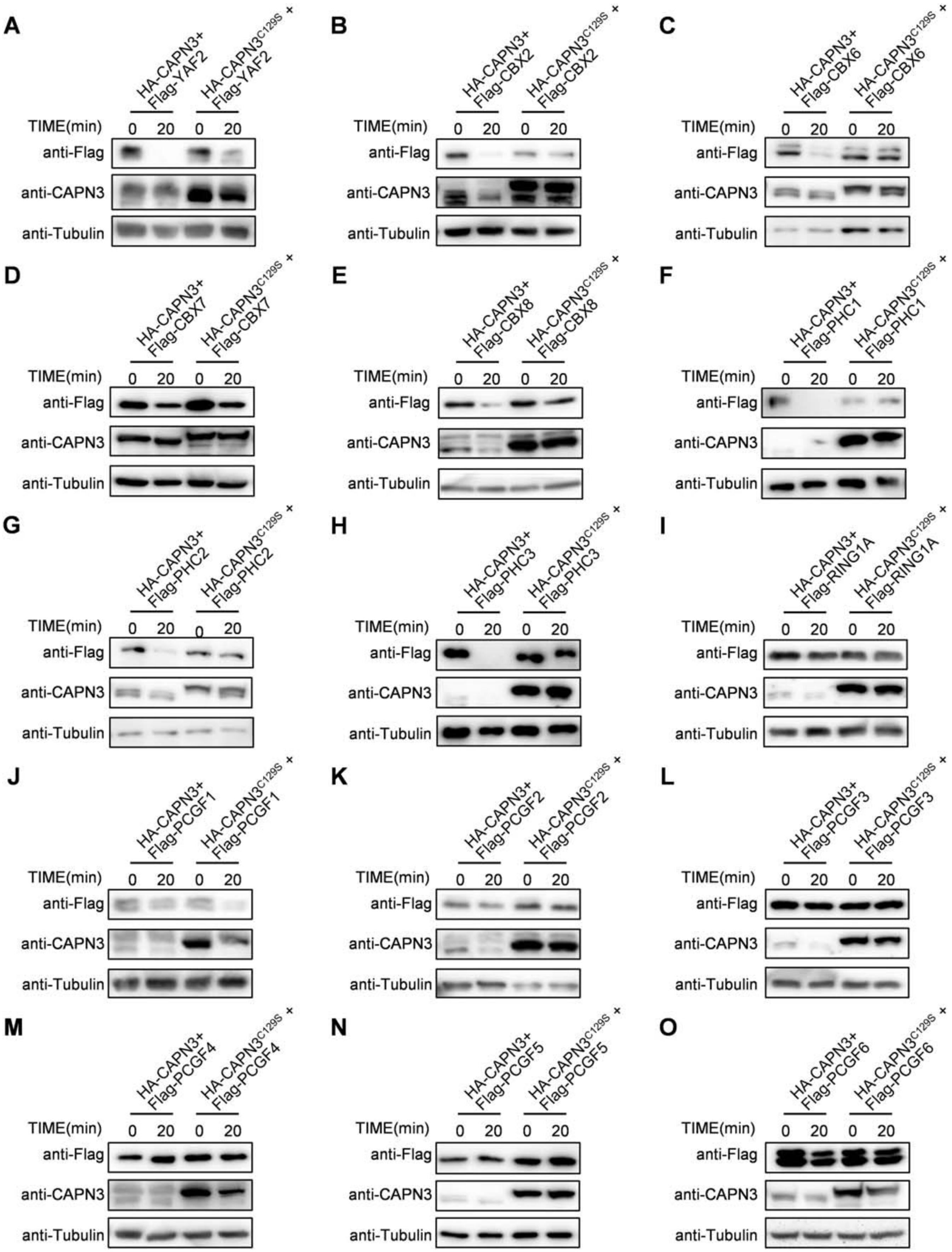
A
HA-CAPN3+ Flag-YAF2
HA-CAPN3C129S + Flag-YAF2
B
HA-CAPN3+ Flag-CBX2
HA-CAPN3C129S + Flag-CBX2
C
HA-CAPN3+ Flag-CBX6
HA-CAPN3C129S + Flag-CBX6
D
HA-CAPN3+ Flag-CBX7
HA-CAPN3C129S + Flag-CBX7
E
HA-CAPN3+ Flag-CBX8
HA-CAPN3C129S + Flag-CBX8
F
HA-CAPN3+ Flag-PHC1
HA-CAPN3C129S + Flag-PHC1
G
HA-CAPN3+ Flag-PHC2
HA-CAPN3C129S + Flag-PHC2
H
HA-CAPN3+ Flag-PHC3
HA-CAPN3C129S + Flag-PHC3
I
HA-CAPN3+ Flag-RING1A
HA-CAPN3C129S + Flag-RING1A
J
HA-CAPN3+ Flag-PCGF1
HA-CAPN3C129S + Flag-PCGF1
K
HA-CAPN3+ Flag-PCGF2
HA-CAPN3C129S + Flag-PCGF2
L
HA-CAPN3+ Flag-PCGF3
HA-CAPN3C129S + Flag-PCGF3
M
HA-CAPN3+ Flag-PCGF4
HA-CAPN3C129S + Flag-PCGF4
N
HA-CAPN3+ Flag-PCGF5
HA-CAPN3C129S + Flag-PCGF5
O
HA-CAPN3+ Flag-PCGF6
HA-CAPN3C129S + Flag-PCGF6
TIME(min) 0 20 0 20
anti-Flag
anti-CAPN3
anti-Tubulin

Figure EV3. CAPN3-mediated proteolysis of PRC1 components.

(**A–O**) Western blot analysis of in vitro CAPN3 proteolysis assays of PRC1 components (**A**) YAF2, (**B**) CBX2, (**C**) CBX6, (**D**) CBX7, (**E**) CBX8, (**F**) PHC1, (**G**) PHC2, (**H**) PHC3, (**I**) RING1A, (**J**) PCGF1, (**K**) PCGF2, (**L**) PCGF3, (**M**) PCGF4, (**N**) PCGF5, (**O**) PCGF6. The incubation times and antibodies used are indicated. These experiments were replicated at least twice.

 © The Author(s)

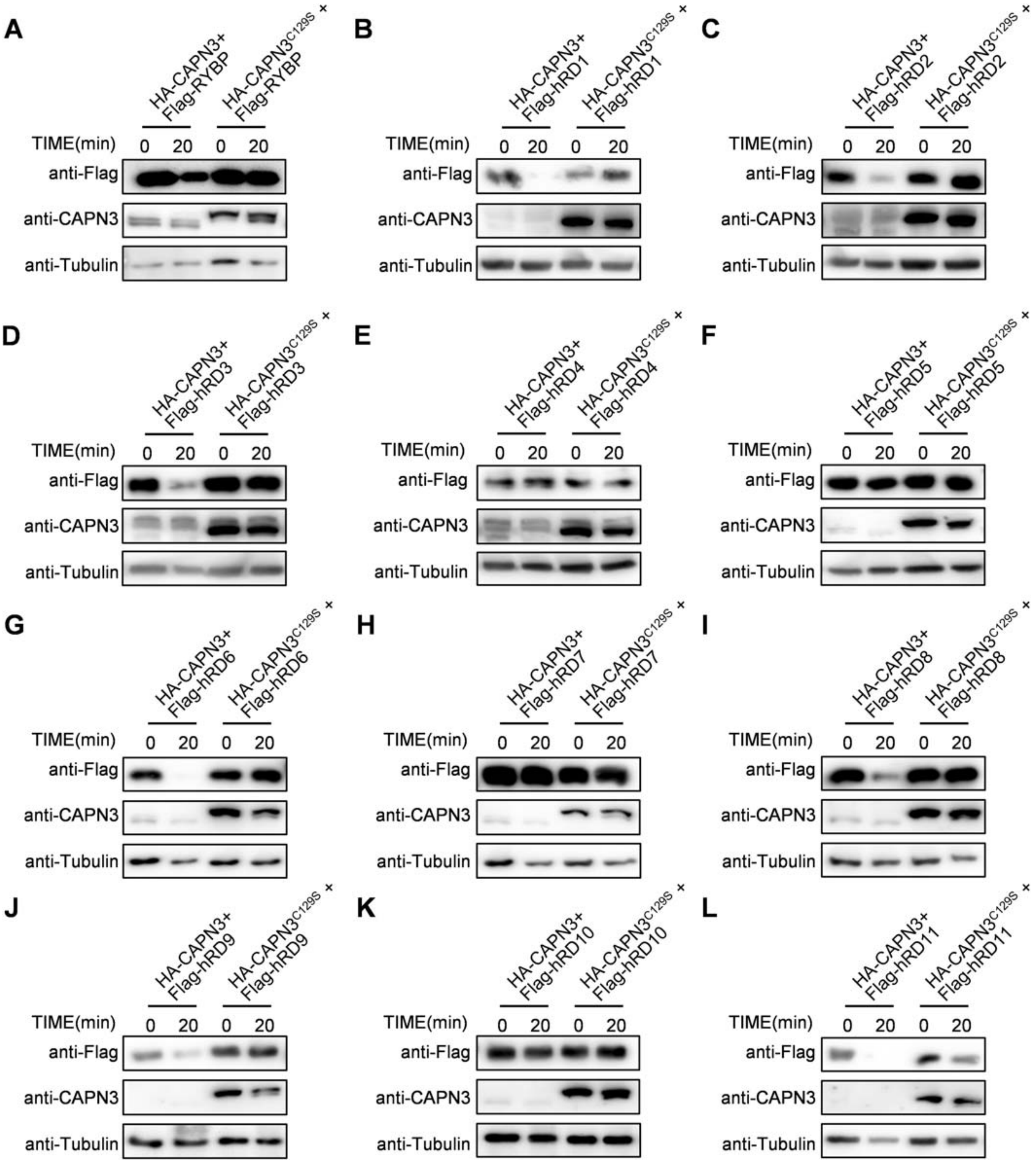

Figure EV4. The 67th to 75th amino acids of RYBP are required for the proteolysis of RYBP by CAPN3.

(**A–L**) Western blot analysis of in vitro CAPN3 proteolysis assays of different amino acids deletion mutants of human RYBP. (**A**) RYBP, (**B**) hRD1, (**C**) hRD2, (**D**) hRD3, (**E**) hRD4, (**F**) hRD5, (**G**) hRD6, (**H**) hRD7, (**I**) hRD8, (**J**) hRD9, (**K**) hRD10, (**L**) hRD11. The incubation times and antibodies used are indicated. These experiments were replicated at least twice.

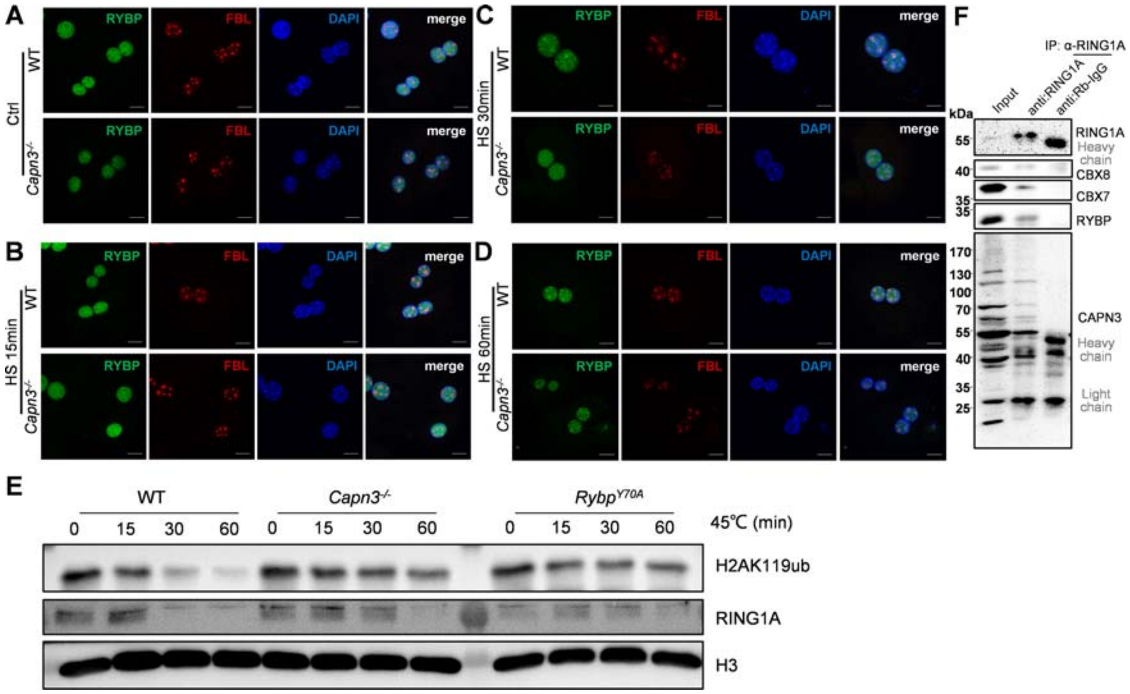

Figure EV5. CAPN3 facilitates nucleolus entry of RYBP and H2AK119ub elimination during stress response.

(**A–D**) Immunostaining of RYBP and FBL (Fibrillarin) (a nucleolus marker) in WT and *Capn3*$^{-/-}$ primary hepatocyte cells after incubated at 45 °C for 0, 15, 30, or 60 min. Scale bars, 10 μm. (**E**) Chromatin fractions were isolated from WT, *RYBP*^{Y70A} homozygous and *Capn3*$^{-/-}$ primary hepatocytes incubated at 45 °C for 0, 15, 30, or 60 min. Western blot analysis was performed with indicated antibodies. This experiment was replicated at least twice. (**F**) Western blot analysis of Input and immunoprecipitation samples from rat liver nuclear protein extracts (without PHx or heat shock). 'Input' contains ~10% of the input cell lysate used for IP. Antibodies used are indicated.

 © The Author(s)
